# Identification and characterization of hirudin-HN, a new thrombin inhibitor, from the salivary glands of *Hirudo nipponia*

**DOI:** 10.7717/peerj.7716

**Published:** 2019-09-30

**Authors:** Boxing Cheng, Fei Liu, Qiaosheng Guo, Yuxi Lu, Hongzhuan Shi, Andong Ding, Chengfeng Xu

**Affiliations:** 1 Institute of Chinese Medicinal Materials, Nanjing Agricultural University, Nanjing, China; 2 School of Biological Sciences, Guizhou Education University, Gui Yang, China; 3 School of Marine and Bioengineering, Yancheng Institute of Technology, Yan Cheng, China

**Keywords:** Leech, Affinity, Salivary glands, Protein structure, Antithrombin, Hirudin-HN

## Abstract

Transcriptome sequencing data (6.5 Gb) of the salivary glands of the haematophagous leech *Hirudo nipponia* was obtained by using the BGIseq-500 platform. After identification and analysis, one transcript (Unigene5370) was annotated to hirudin HV3 from *Hirudo medicinalis* with an *e*-value of 1e-29 and was named hirudin-HN. This transcript was a new thrombin inhibitor gene belonging to the proteinase inhibitor I14 (hirudin) family. Hirudin-HN, with a 270-bp cDNA, encodes an 89-aa protein containing a 20-aa signal peptide. The mature hirudin-HN protein contains the typical structural characteristics of hirudin, e.g., three conserved disulfide bonds and the PKP and DFxxIP motifs. Proteins (*Hir* and *M-Hir*) were obtained via prokaryotic expression, and the mature hirudin-HN protein was shown to have anticoagulant activity and thrombin affinity by using the chromogenic substrate S2238 and surface plasmon resonance (SPR) interaction analysis, respectively. The N-terminal structure of the mature hirudin-HN protein was shown to be important for anticoagulant activity by comparing the activity and thrombin affinity of *Hir* and *M-Hir*. The abundances of Hirudin-HN mRNA and protein were higher in the salivary glands of starving animals than in those of feeding or fed leeches. These results provided a foundation for further study on the structure-function relationship of hirudin-HN with thrombin.

## Introduction

*Hirudo nipponia*, which feeds mainly on the blood of mammals or frogs, is widely distributed in China (Yang, 1996), and this leech is listed as an animal raw material for Chinese medicinal materials (Commission, 2015). The use of leeches in medicine has been documented, first appearing in *Farmer’s Classic of Materia Medica*, which was written in the late Han dynasty (Zhang et al., 2013). The main pharmacological effects of leeches include anticoagulation (Müller et al., 2016), thrombolytic (Baskova et al., 2001), and anti-inflammatory effects (Nei et al., 2014) and improvement of cardiovascular and cerebrovascular diseases (Liu et al., 2016). The underlying pharmacological components are mainly secreted by the salivary glands of leeches. Therefore, in recent years, transcriptome sequencing analysis has been carried out on the salivary glands of leeches, and the pharmacological components have been screened and subjected to evolutionary analysis (Min, Sarkar & Siddall, 2010; Kvist et al., 2014; Kvist et al., 2016; Siddall, Brugler & Kvist, 2016). These components are divided into four groups based on their different pharmacological effects. The first group is the anticoagulation group, members of which can inhibit thrombin or factor Xa activity; this group includes hirudins, hirudin-like factors, antistasin (Tuszynski, Gasic & Gasic, 1987), therostasin (Chopin et al., 2000), guamerin (Kim et al., 2008), haemadin (Strube et al., 1993), and bufrudin (Electricwala et al., 1993). The second group is the platelet aggregation inhibitor group, consisting of saratin (White et al., 2007), decorsin (Seymour et al., 1990), ornatins (Mazur et al., 1991), calin and leech antiplatelet protein (LAPP) (Schaffer et al., 1993). The third group is the fibrin degradation group (destabilase I and destabilase II), members of which have the capacity to degrade fibrin (Zavalova et al., 1996). The fourth group is the antimicrobial peptide (AMP) group, which includes destabilase I, destabilase II and hirudomacin (Ding et al., 2019); members of this group can inhibit microbial growth.

Hirudin is the most powerful thrombin-specific inhibitor identified to date and is a representative pharmacologically active substance in leeches (Markwardt, 1994). In the Pharmacopoeia of the People’s Republic of China, antithrombin activity is the only standard for determination of leech quality (Commission, 2015). Therefore, detection of hirudin and determination of available hirudin content are important standards for testing of leech efficacy. In modern studies, the structural characteristics of hirudin have been studied, and the mechanism underlying the inhibition of thrombin by hirudin has been elucidated (Rydel et al., 1990; Rydel et al., 1991; Vitali et al., 1992; Karshikov et al., 1992). Natural hirudin is composed of 65 or 66 amino acids and has a molecular weight of approximately 7,000 Da (Dodt et al., 1986). The N-terminal peptide chain of hirudin contains three conserved intramolecular disulfide bonds, leading to the formation of a stable core structure by the N-terminal peptide chain (Dodt et al., 1985; Chang, Schlaeppi & Stone, 1990). The first three amino acids at the N-terminus have special significance for hirudin, as these residues block the active sites of thrombin (Betz, Hofsteenge & Stone, 1992). When researchers mutated these three amino acids, the anticoagulant activity decreased significantly (Huang et al., 2014). The peptide chain contains a special Pro-Lys^47^-Pro (PKP) sequence, the main function of which is to maintain the stability of hirudin molecules and guide the binding of these molecules to thrombin in the correct direction (Braun et al., 1988; Betz, Hofsteenge & Stone, 1992). The C-terminus contains DFxxIP motifs that block the fibrinogen recognition site on thrombin (Müller et al., 2017).

Hirujins A and B, which inhibit thrombin activity, have also been identified in *H. nipponia*, but these molecules do not have the characteristics of hirudin (Hong, 1999). Leech granulin, which is similar to granulin or epithelin at the N-terminus, was purified from *H. nipponia* and was classified as a thrombin inhibitor (Hong & Kang, 1999). Many other active pharmacological components have been isolated from *H. nipponia*. Guamerin is a human leukocyte elastase inhibitor with an inhibition constant of 8. 1 ×10^−14^ M. Piguamerin, which was purified by sequential chromatography, is a strong inhibitory effect on plasma kallikrein, tissue kallikrein and trypsin (Kim & Kang, 2010). Hirudomacin, an antimicrobial protein that can kill *Staphylococcus aureus* and *Bacillus subtilis*, was cloned from a salivary gland cDNA library of *H. nipponia* (Ding et al., 2019). The biological behaviours of *H. nipponia*, such as oxygen consumption (Luo et al., 1998), reproductive performance (Ping et al., 2015), growth and feeding (Lu et al., 2017), have been thoroughly studied.

Transcriptome sequencing of the salivary glands of many leeches has been performed. Many studies have investigated the anticoagulant activity and the underlying mechanism by isolating anticoagulant proteins and cloning the associated genes from leeches. However, anticoagulant transcripts have rarely been screened by transcriptome sequencing of salivary glands from *H. nipponia*. Based the results of this study, the salivary glands of *H. nipponia* contain many transcripts corresponding to proteins with putative pharmacologically active substances, and further study of hirudin-HN showed a potent and specific inhibitory effect on thrombin. To the best of our knowledge, this is the first report about a hirudin-type protein from *H. nipponia*.

## Materials & Methods

### Leech experiments and RNA extraction

Leeches (*H. nipponia*) were kept at the Institute of Chinese Medicinal Materials of Nanjing Agricultural University. The leeches had not eaten for at least a month before the experiment. Healthy leeches with an average weight of approximately 0.5 g (0.5 ± 0.09 g) were selected for the experiment. Leeches were fed with sufficient fresh pig blood that was poured into pig gut casing during the experiment. During three periods of blood meal feeding (unfed group (UFG): not fed, feeding group (FIG): 20 min after initiation of blood meal feeding, and fed group (FG): 24 h after satiation), the salivary glands were aseptically collected as described previously (Müller et al., 2017). The aforementioned tissues were stored in RNAlater (Qiagen, GER) according to the manufacturer’s specifications at −80 °C.

Total RNA from each group (UFG, FIG and FG) was extracted using the TaKaRa MiniBEST Universal RNA Extraction Kit (TaKaRa, JPN) according to the manufacturer’s instructions. The quality of the total RNA was determined using a Nano-300 microspectrophotometer (Allsheng, CHN). The total RNA of salivary glands tissue (SA) from UFG was reverse transcribed to cDNA using the PrimeScript™ II 1st Strand cDNA Synthesis Kit (TaKaRa, JPN) for cloning of anticoagulant genes. The total RNAs of UFG, FIG and FG were reverse transcribed to cDNA by using the PrimeScript™ RT Reagent Kit with gDNA Eraser (TaKaRa, JPN) for qPCR. Then, the cDNAs were stored at −20 °C.

All handling of *H. nipponia* was conducted in accordance with the Guidelines for the Care and Use of Animals for Scientific Purposes recommended by the Institutional Animal Care and Use Committee (IACUC) of Nanjing Agricultural University, China. The IACUC specifically approved this study within the project “The artificial culture of medicinal leeches” (approval number NAU (F)-17-041).

### cDNA library construction, BGISEQ-500 sequencing and *de novo* assembly

Three groups (UFG, FIG and FG) of qualified total RNA were mixed as sequencing samples. According to the flow chart of the experimental procedure (Fig. S1), the total RNA from the salivary gland of *H. nipponia* was used to prepare a single-stranded circular DNA library. Then, the prepared library was sequenced using the BGISEQ-500 platform (BGI) at the Beijing Genomics Institute.

Reads with low quality, adaptor contamination or high levels of unknown bases (N) were removed before data analyses to ensure reliability of the results. After filtering, the remaining reads were termed “clean reads” and stored in FASTQ format (Cock et al., 2009). We used Trinity to perform *de novo* assembly with clean reads (PCR duplicates were removed to improve efficiency). TGICL was used to cluster transcripts and remove redundancy, and unigenes were finally obtained. Data were submitted to the NCBI Sequence Read Archive under accession number SRP151118.

### Sequence annotation

Unigenes were annotated with NT using Blastn; Blastx and Diamond (Buchfink, Xie & Huson, 2015) were used to annotate unigenes with the NR, KOG, KEGG and SwissProt databases; Blast2GO (Conesa, Terol & Robles, 2005) and NR were used for GO annotation; and InterPro Scan5 (Quevillon et al., 2005) was used for InterPro annotation. Transcripts of anticoagulant proteins were screened from the above annotation results, and a cut-off *e*-value of 1e−5 was used. Clean reads were compared to UniGene using Bowtie2 (Langmead & Salzberg, 2012), and the gene expression levels were calculated using RSEM (Li & Dewey, 2011).

### Cloning of the cDNA encoding hirudin-HN

Unigene5370 was screened for further analysis because this transcript from the above salivary gland transcriptome database of *H. nipponia* shared the highest homology with the hirudin variant HV3 (ALA22935.1), and the *e*-value was 1e−29; we named the Unigene5370 transcript hirudin-HN. Amplification primers were designed using SnapGene ver. 3.2.1 based on the open reading frame (ORF) of hirudin-HN and synthesized by Nanjing GenScript Biotechnology Company (Table 1). The cDNA for cloning was used as a template for the amplification system. The amplification procedure and reaction system were performed according to the manufacturer’s instructions for Prime STAR GXL DNA Polymerase. The PCR products were verified by agarose electrophoresis and then purified. The recovered products were connected to the pEASY-Blunt vector and transformed into DH5 *α* competent cells. The positive clones were sent to GenScript for sequencing.

**Table 1:** Primers used for cloning and qPCR.

Primer	Sequence (5′–3′)	Application
Hirudin-HN-F	ATGTTCTCTCTGAAGCTATTTCTTG	Cloning
Hirudin-HN-R	TCATTTATCGTAGTCTTCAATTGGG	Cloning
qHirudin-HN-F	GCGGAGGCCGGTTGGAATTA	qPCR
qHirudin-HN-R	CGGTGAAACTGCGCCAATCG	qPCR
β-Actin-F	ATTGGGCAGATCGTGAGC	qPCR
β-Actin-R	GAAGTGGATGCGAGGGTAG	qPCR

### Bioinformatic analysis of hirudin-HN

Based on the cloned sequence, the full-length amino acid sequence of the hirudin-HN protein was predicted by using ORFfinder (https://www.ncbi.nlm.nih.gov/orffinder/). Protein signal peptides were predicted using the SignalP 3.0 server (http://www.cbs.dtu.dk/services/SignalP-3.0/). Prediction of the physicochemical properties of the hirudin-HN protein after removal of signal peptides was performed by the ExPASy ProtParam tool (https://web.expasy.org/protparam/). Hydropathicity analysis was performed using the ExPASy ProtScale tool (https://web.expasy.org/protscale/). Functional and classification analyses of proteins were performed using InterPro ver. 73 (http://www.ebi.ac.uk/interpro/). Feature prediction for the secondary structure of hirudin-HN was performed using Predict Protein 2013 (https://www.predictprotein.org). Protein BLAST (https://blast.ncbi.nlm.nih.gov/Blast.cgi) was performed based on the hirudin-HN amino acid sequence, and the amino acid sequences were aligned using Jalview ver. 2.10.5. Evolutionary relationships were deduced by the neighbour-joining method, and a phylogenetic tree was constructed using MEGA ver. 10.0.5.

### Prokaryotic expression of the hirudin-HN protein

Based on the gene sequencing results, codon optimization for prokaryotic expression was performed after deletion of the signal peptide, and gene synthesis was carried out based on the expression construct (M–hirudin-HN–His tag, *M-Hir*). The synthesized gene fragment was linked to the pET30a expression vector (GenScript, China). Then, the recombinant plasmid was transformed into *Escherichia coli* BL21(DE3). When the OD_600_ value of the bacterial culture reached 0.6–0.8, isopropyl 1-thio-β-D-galactopyranoside (IPTG) at final concentration of 0.5 mM was added, and the culture was incubated for 16 h at 120 rpm and 16 °C. The induced bacterial cells were collected by centrifugation (4,000 rpm for 10 min), and the sediment was resuspended in PBS (pH 7.4). The samples were sonicated on ice (300 W, 6 min, sonicate for 3 s and rest for 2 s), and the supernatant was collected by centrifugation at 4 °C (12,000 rpm, 10 min). The target protein was purified using a Ni-NTA-Sefinose column (SangonBiotech, Shanghai, China). The protein purification system used an imidazole gradient (0–500 mM) as the target protein eluent. The eluent was collected and stored at −80 °C. Protein expression was detected using 15% SDS-PAGE. The purified band of *M-Hir* was cut out and detected by peptide mass fingerprinting (PMF).

A second expression construct (Trx tag–His tag–enterokinase site–hirudin-HN, *Hir*) was implemented to investigate the effect of N-terminal “Met” residues on the recombinant protein activity, and a recombinant plasmid was constructed with pET-32a (Zoonbio Biotechnology, Jiangsu, China). Then, the recombinant plasmid was transformed into *E. coli* BL21(DE3). Prokaryotic expression of the fusion protein was performed according to the procedure described for first expression construct. The fusion protein was excised using enterokinase (Beyotime, China) on a column at 4 °C for 16 h. The column was washed with washing buffer (20 mM Tris-HCl, 20 mM imidazole, 0.15 M NaCl, pH 8.0). Fluid was collected and used a buffer (20 mM Tris-HCl, 0.15 M NaCl, 5% glycerol, pH 8.0) for dialysis. Protein expression and cleavage of the fusion protein were detected using 15% SDS-PAGE. The purified band of the fusion protein (*Hir*) was cut out and detected by PMF.

### Antithrombin activity assay

The antithrombin activity of the protein was assayed using the chromogenic substrate S2238. The protein concentration of the samples was detected using the BCA Protein Assay Kit (SangonBiotech, Shanghai, China). As a first step, a standard curve of the association between thrombin content and absorbance was prepared. Thrombin, at a range of contents (0–0.25 U, 10 U/mL, bovine alpha-thrombin; Shanghai YuanyeBio-Technology), was incubated with 10 µL of chromogenic substrate S2238 (5 mM, Aglyco, China) at 37 °C for 2 min in a 96-well plate (the reaction system was supplemented with 0.9% NaCl to a volume of 35 µL). Then, 50 µL of 50% acetic acid was used to terminate the reaction, and the reaction system was supplemented with distilled water to a final volume of 250 µL. Then, the OD_405_ values of the reaction systems with different thrombin concentrations were measured by a microplate assay (BioTek, Winooski, VT, USA). The second step was detection of the antithrombin activity of the target protein. Twenty microlitres of a moderate concentration of the sample protein and 20 µL of normal saline as a control were each mixed with 30 µL of thrombin (10 U/mL) at 37 °C for 5 min in a 96-well plate. Then, 10 µL of the chromogenic substrate S2238 was added, and the plate was incubated at 37 °C for 2 min, followed by addition of 50 µL of 50% acetic acid as the reaction terminator. The volume of the reaction system was increased to 250 µL with distilled water, and the absorbance of each reaction was measured at 405 nm. Finally, the standard curve was used to calculate the antithrombin activity of the protein samples.

### Surface plasmon resonance-based interaction analysis

Surface plasmon resonance (SPR)-based interaction analysis of the sample protein and thrombin was performed using a Biacore 8K instrument (GE Healthcare, Wauwatosa, WI, USA). Sample protein was diluted to 10 µg/mL in 10 mM sodium acetate (pH 4.0) and injected over 420 s at 10 µL/min with approximately 5,000–6,000 RU. Sample protein was immobilized via amine coupling to a series S CM5 chip (GE, USA). Thrombin was diluted in running buffer (PBS, 0.05% P20 (GE, USA)).

Thrombin plated in a 9-point dose response with a compound concentration of 0 as a reference, and the highest compound concentration used was 10 µM (run parameters: 30 µL/min, high-performance injection and data collection at 10 Hz, temperature at 250 °C, association time of 120 s and dissociation time of 600 s). Binding curves are shown, and the affinity constants (K_D_ values) were calculated by BIA evaluation 4.1 software.

### Analysis of hirudin-HN mRNA expression by qPCR

The cDNA for qPCR served as the template to study the expression pattern of hirudin-HN. Samples were examined by the SYBR Green qPCR system (QuantStudio™ 7 Flex; Applied Biosystems) with the SYBR^®^ Premix Ex Taq™ -Tli RNaseH Plus Kit (TaKaRa, JPN). The GenScript Real-time PCR (TaqMan) Primer Design tool http://www.genscript.com/tools/real-time-pcr-tagman-primer-design-tool) was used to design the primers, which were then synthesized by Nanjing GenScript Biotechnology Company. Relative expression was calculated by the 2^−ΔΔ*CT*^ method using the reference gene *β*-actin.

### Production of polyclonal antibodies

The recombinant protein *M-hir* was purified for the production of polyclonal antibodies in New Zealand white rabbits according to previously reported methods (Yang et al., 2007). The first immunization of each rabbit was with 0.2 mg of antigen and an iso-volume of Freund’s complete adjuvant. The second, third and fourth immunizations consisted of 0.1 mg of antigen and an of iso-volume Freund’s incomplete adjuvant. After the third immunization, 1 ml of venous blood was collected from the ears at 7 d after each immunization to assess antiserum titres. The antiserum titres fulfilled the experimental requirements, and whole blood was collected from the carotid arteries of the rabbits. Rabbit serum was diluted at 1:1,000 and used as a primary antibody. The secondary antibody (goat anti-rabbit IgG (H + L), horseradish peroxidase (HRP)-labelled) was diluted 1:8,000. The OD value was measured at 450 nm. The post-purification antibody titres were A ≥512 K and B ≥512 K.

### Analysis of hirudin-HN protein expression by western blotting

The total proteins of UFG, FIG and FG were extracted using the Tissue or Cell Total Protein Extraction Kit (SangonBiotech, Shanghai, China), and the protein concentration of each sample was determined by the BCA Protein Assay Kit (SangonBiotech, Shanghai, China). The protein concentration of each sample was adjusted to five µg/µL. As a primary antibody, the polyclonal antibody was diluted 1:2,500 with 5% BSA-TBST. Goat anti-rabbit IgG (H + L) HRP was diluted in 5% NFDM-TBST (1:8,000) as a secondary antibody. After being washed, the membrane was treated with chemiluminescence reagent (ECL) for 5 min. Finally, based on the effect on the photographic film, the exposure and development time were adjusted. Quantification of the protein was performed using ImageJ software. Beta-actin was used as a control.

### Homology modelling and docking simulation

The *Hir* structure was modelled using the MODELLER program with a crystal structure as a template (PDB ID: 2PW8) (Marti-Renom et al., 2000). The sequences were aligned using Clustal Omega with a sequence identity of 57.8% (Madeira et al., 2019). An additional N-terminal residue, methionine, was linked to the *Hir* model to build the *M-Hir* model. These two structures were further refined using AMBER force field for 5,000 steps (Case et al., 2005). Protein-Protein Docking (ZDOCK) was used to investigate the interaction between *Hir*/*M-Hir* and thrombin (PDB ID: 1HRT) (Pierce et al., 2014). The predicted binding modes are similar to the hirudin thrombin complexes (PDB ID: 4HTC and 1HRT). The final complex models were tweaked and rebuilt on the crystal complex (PDB ID: 1HRT) using the Pymol tool. They were refined using AMBER force field for 5,000 steps.

## Results

### Characteristics of the assembled salivary transcriptome

The transcriptome of *H. nipponia* salivary glands was sequenced by the BGIseq-500 platform to obtain 6.5 Gb of data. A total of 41,895 unigenes were obtained after assembly and removal of redundancy. The values for total length, average length, N50 and GC content obtained were 46,785,456 bp, 1,116 bp, 1,968 bp and 42.12%, respectively. The transcriptome data were submitted to the NCBI Sequence Read Archive under accession number SRP151118. Then, the unigenes were compared to seven functional databases for annotation. Finally, 24,723 unigenes (NR: 59.01%), 7,542 unigenes (NT: 18.00%), 20,526 unigenes (SwissProt: 48.99%), 19,303 unigenes (KOG: 46.07%), 19,862 unigenes (KEGG: 47.41%), 7,549 unigenes (GO: 18.02%) and 19,707 unigenes (InterPro: 47.04%) received annotation. Moreover, 27,936 coding sequences (CDS) were detected using Transdecoder. In addition, 8,931 SSRs were detected in 6,026 unigenes, and unigenes encoding 3,969 transcription factors were predicted.

### Predicted anti-coagulation proteins in the *H. nipponia* leech salivary gland transcriptome

Transcripts corresponding to proteins with putative antithrombotic activity were found in the transcriptome of the salivary glands of *H. nipponia* at an *e*-value of 1e− 5 or below (Table 2). These transcripts are classified as thrombin inhibitors, platelet aggregation inhibitors, factor Xa inhibitors, and thrombolytics according to different mechanism of action. For example, the transcripts included hirudin variant HV3 from *Hirudo medicinalis*, hirudin-HM1 from *Poecilobdella manillensis*, guamerin from *P. manillensis*, saratin from *Haementeria officinalis*, destabilase I from *H. medicinalis*, and disintegrin from *Crassostrea gigas*. Notably, Unigene5370 was annotated as the hirudin variant HV3 of *H. medicinalis* (ALA22935.1), and the *e*-value reached 1e−29. In this study, the *e*-value of Unigene5370 was the best among the hirudin transcripts, so this transcript was selected for further study.

**Table 2:** Transcripts with putative antithrombotic activity.

Transcript no.	Best match to the NR database	*E*-value	Sequence ID	Putative activity	FPKM
Unigene5370	Hirudin variant HV3 [*Hirudo medicinalis*]	1e−29	ALA22935.1	Thrombin inhibitor (Müller et al., 2016)	2,951.95
Unigene3091	Hirudin-HM1 [*Poecilobdella manillensis*]	2e−14	Q07558.1	Thrombin inhibitor (Scacheri et al., 1993)	12,283.46
Unigene12407	Guamerin [*Hirudo nipponia*]	1e−31	P46443.1	Elastase inhibitor (Jung et al., 1995)	54.56
Unigene19293	Guamerin [*Poecilobdella manillensis*]	1e−17	ARW78052.1	Elastase inhibitor	64.61
Unigene2825	Saratin [*Haementeria officinalis*]	2e−42	2K13_X	Inhibits platelet aggregation (Gronwald et al., 2008)	3,734.84
Unigene379	Destabilase I [*Hirudo medicinalis*]	3e−55	AAA96144.1	Fibrinolytic activity (Zavalova et al., 1996)	1,965.95
CL1923.Contig5	Destabilase I [*Hirudo medicinalis*]	9e−73	AAA96144.1	Fibrinolytic activity (Zavalova et al., 1996)	424.4
CL359.Contig2	Disintegrin [*Crassostrea gigas*]	2e−28	EKC29034.1	Potential inhibitor of platelet aggregation (Zhang et al., 2012)	493.11
CL2866.Contig1	Disintegrin [*Crassostrea virginica*]	1e−46	XP_022324395.1	Potential inhibitor of platelet aggregation	1,655.56
Unigene17477	Disintegrin [*Mizuhopecten yessoensis*]	7e−49	XP_021370783.1	Potential inhibitor of platelet aggregation	1,571.64
CL2798.Contig2	Disintegrin [*Crassostrea gigas*]	5e−48	EKC29034.1	Potential inhibitor of platelet aggregation (Zhang et al., 2012)	516.9
Unigene25705	Disintegrin [*Homo sapiens*]	2e−32	XP_019926910.1	Potential inhibitor of platelet aggregation	974.79
Unigene2342	Peptidase inhibitor 16 [*Trichinella papuae*]	1e−34	KRZ76828.1	Inhibits trypsin; potential cardiac function (Korhonen et al., 2016)	577.12
Unigene3360	Serpin [*Thioploca ingrica*]	4e−64	WP_045475444.1	Coagulation cascade regulation	505.57
CL4396.Contig2	Serpin [*Ailuropoda melanoleuca*]	3e−61	XP_002926410.1	Coagulation cascade regulation	519.11

**Notes.**

The assembled sequences of unigenes and of the contigs listed in Table S1

### Structure of the hirudin-HN protein

A 270-bp cDNA (NCBI NO. MK947218) encodes the 89-aa hirudin-HN, containing a 20-aa signal peptide at the N-terminus and a 69-aa mature protein sequence. After removal of the signal peptide, the predicted molecular weight of the mature hirudin-HN was 7678.48 Da, and the theoretical pI was 4.84. The total number of positively charged residues (Arg + Lys) was 9, and the number of negatively charged residues (Asp + Glu) was 13. The aliphatic index was 53.62, and the grand average of hydropathicity (GRAVY) was −1.125. ProtScale results indicated the greatest hydrophilicity and hydrophobicity of the mature hirudin-HN protein to occur at Pro^48^ and Glu^17^, respectively.

**Figure 1:**
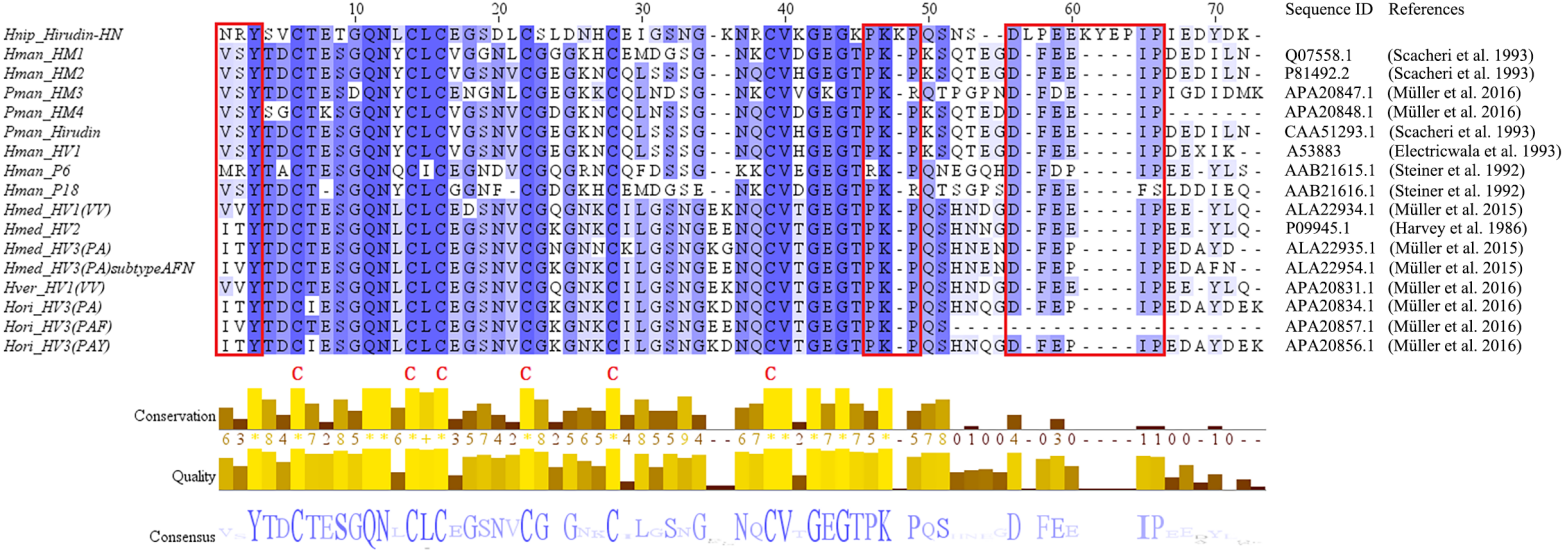
Multiple sequence alignments of the primary structures of hirudin-HN, HM1-4, hirudin, HV1, P6, P18, HV1(VV), HV2, HV3(PA), HV3(PA) subtype AFN, HV1(VV), HV3(PAF) and HV3(PAY). The three N-terminal amino acids and the PKP and DFxxIP motifs are shownin red boxes. The six conserved Cys residues are marked with a red “C” under each sequence. Hnip, *Hirudo nipponia*; Hman, *Hirudinaria manillensis*; Pman, *Poecilobdella manillensis*; Hmed, *Hirudo medicinalis*; Hman, *Hirudinaria manillensis*; Hver, *Hirudo verbana*; Hori, *Hirudo orientalis*.

**Figure 2:**
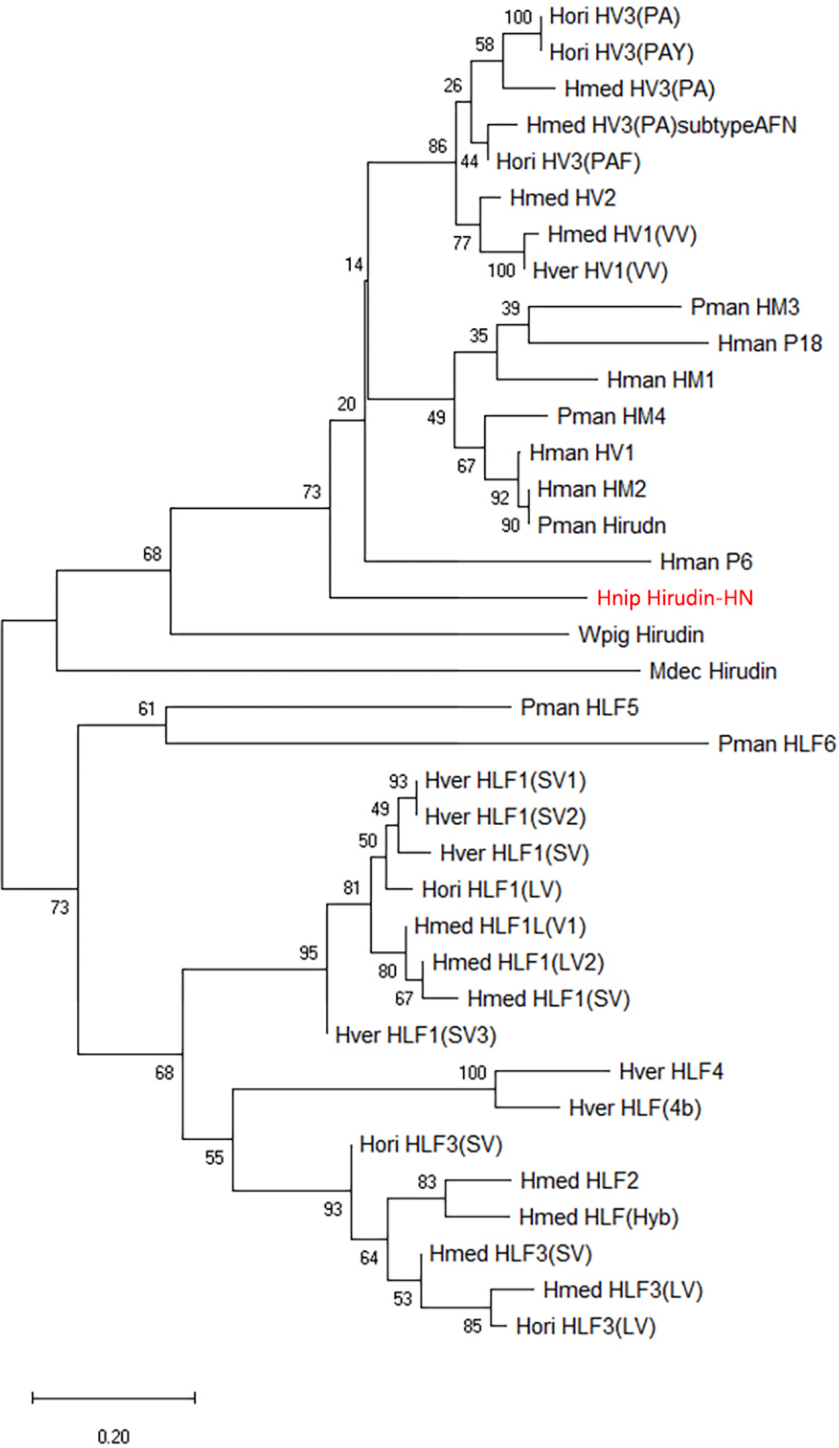
A tree of the mature hirudin-HN amino acid sequences. Scale bars indicate substitutions per site. Hori, *Hirudo orientalis*; Hmed, *Hirudo medicinalis*; Hver, *Hirudo verbana*; Pman, *Poecilobdella manillensis*; Hman, *Hirudinaria manillensis*; Hnip, *Hirudo nipponia*; Wpig, *Whitmania pigra*; Mdec, *Macrobdella decora*.

The primary structure of the mature hirudin-HN protein exhibits the highest similarity with hirudin variant HV3, which belong to the proteinase inhibitor I14 (hirudin) family (IPR000429). Based on the secondary structure prediction, there were three pairs of disulfide bonds, namely, C6-C14, C16-C28 and C22-C38, and a disordered region from residues 39 to 69. The PKP and DFxxIP structural motifs of hirudin were also identified in protein sequences by multiple sequence alignment (Fig. 1). The protein sequence of mature hirudin-HN was used to construct a phylogenetic tree of reported hirudins and hirudin-like factors (HLFs), and hirudin and HLFs were clearly separate from each other as sister groups (Fig. 2). Hirudin-HN could be observed in the hirudin group in the phylogenetic tree. Therefore, hirudin-HN could be classified as a hirudin in terms of both structural characteristics and phylogenetic evolution.

### Prokaryotic expression of the hirudin-HN protein

**Figure 3:**
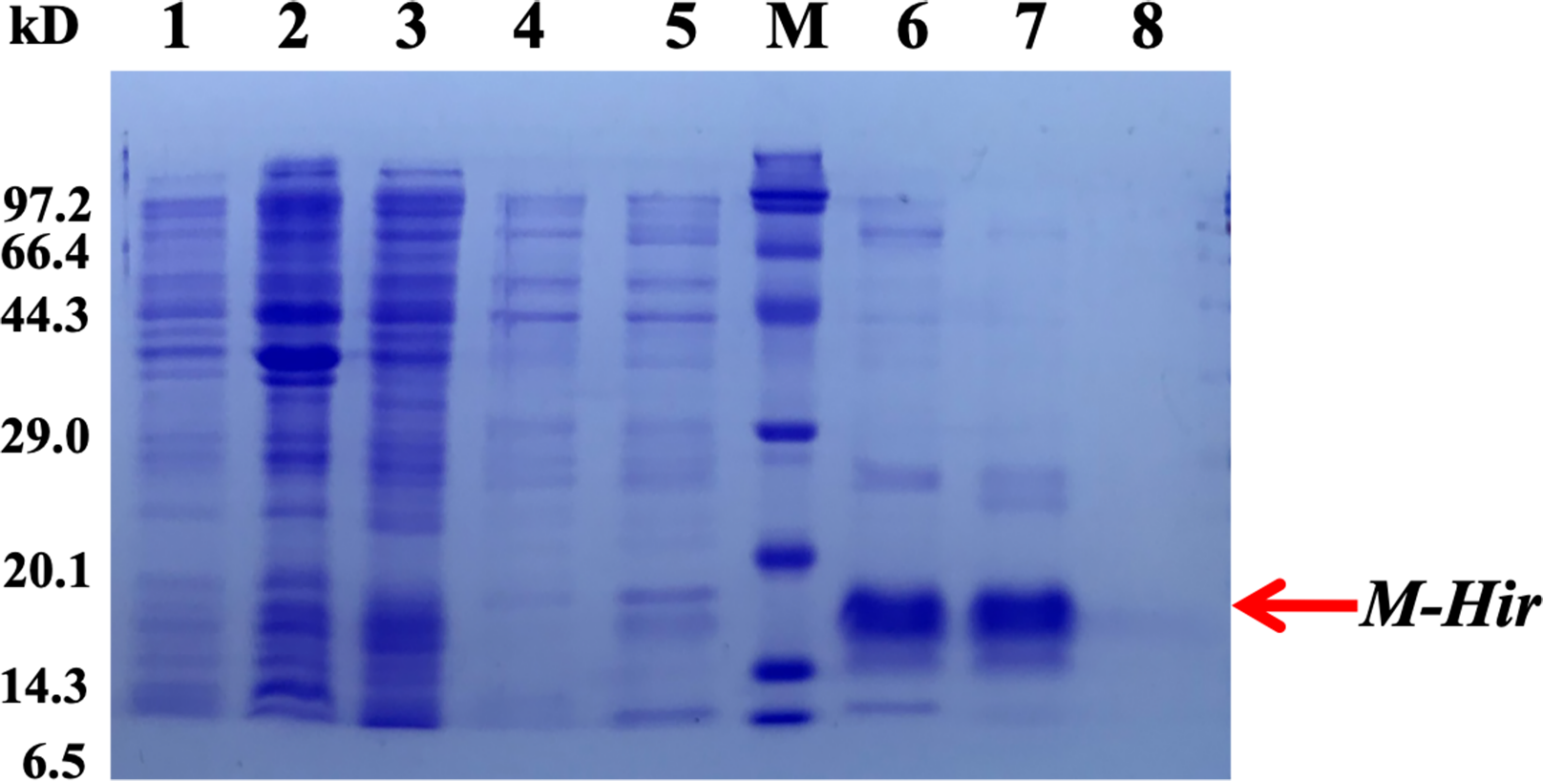
Expression and purification of the mature *M-Hir* protein. M: protein marker; lane 1: CK (without IPTG); lane 2: precipitate after pET30a-*M-Hir* induction; lane 3: supernatant after pET-30a-*M-Hir* induction; lane 4–8: imidazole gradient-eluted *M-Hir* protein (20, 50, 100, 250 and 500 mM).

**Figure 4:**
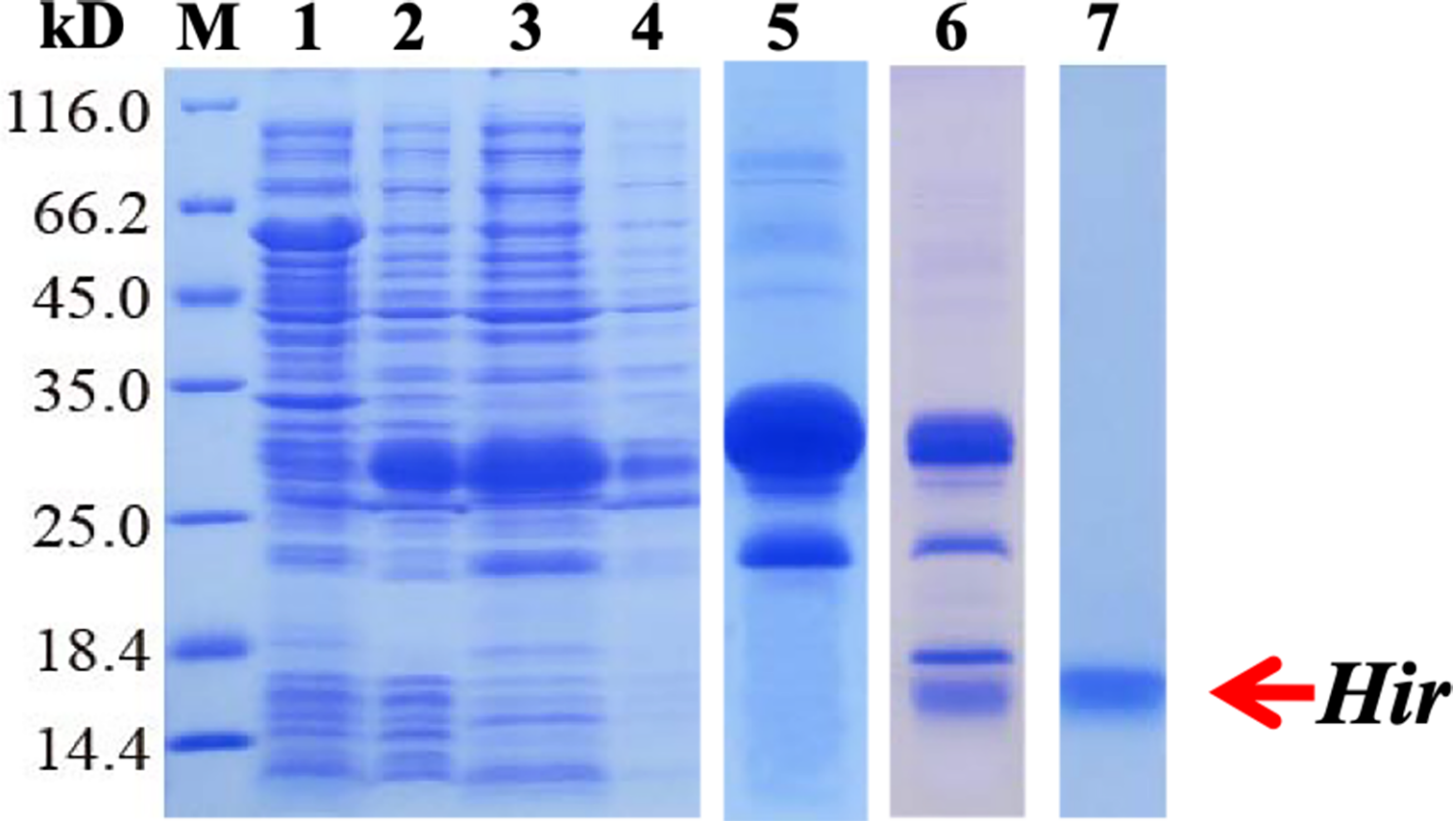
Expression, digestion and purification of the mature *Hir* protein. M: protein marker; lane 1: CK (without IPTG); lane 2: whole-cell lysate after pET32a-*Hir* induction; lane 3: supernatant after pET32a-*Hir* induction; lane 4: precipitate after pET32a-*Hir* induction; lane 5: eluted *Hir* fusion protein; lane 6: fusion protein digested by enterokinase; lane 7: purified *Hir*.

To further study the properties of hirudin-HN, two prokaryotic expression constructs were used to express *M-Hir* and *Hir*. After induction of expression, the bacteria harbouring the *M-Hir* expression construct were collected, and a culture without IPTG was used as the control. After sonication, centrifugation and purification, *M-Hir* was successfully obtained in the supernatant, as detected by 15% SDS-PAGE (Fig. 3). PMF results demonstrated the correct aa sequence for *M-Hir* (File S2). The *M-Hir* shown in lane 7 was used for subsequent experiments. By using the abovementioned method for induction of expression, the *Hir* fusion protein was also successfully expressed (Fig. 4). PMF results demonstrated the correct aa sequence for the *Hir* fusion protein (File S3). The mature *Hir* protein was successfully obtained, as shown in lane 7 (Fig. 4), by on-column enzyme digestion and dialysis.

### Performance assay of the *M-Hir* and *Hir* proteins

**Figure 5:**
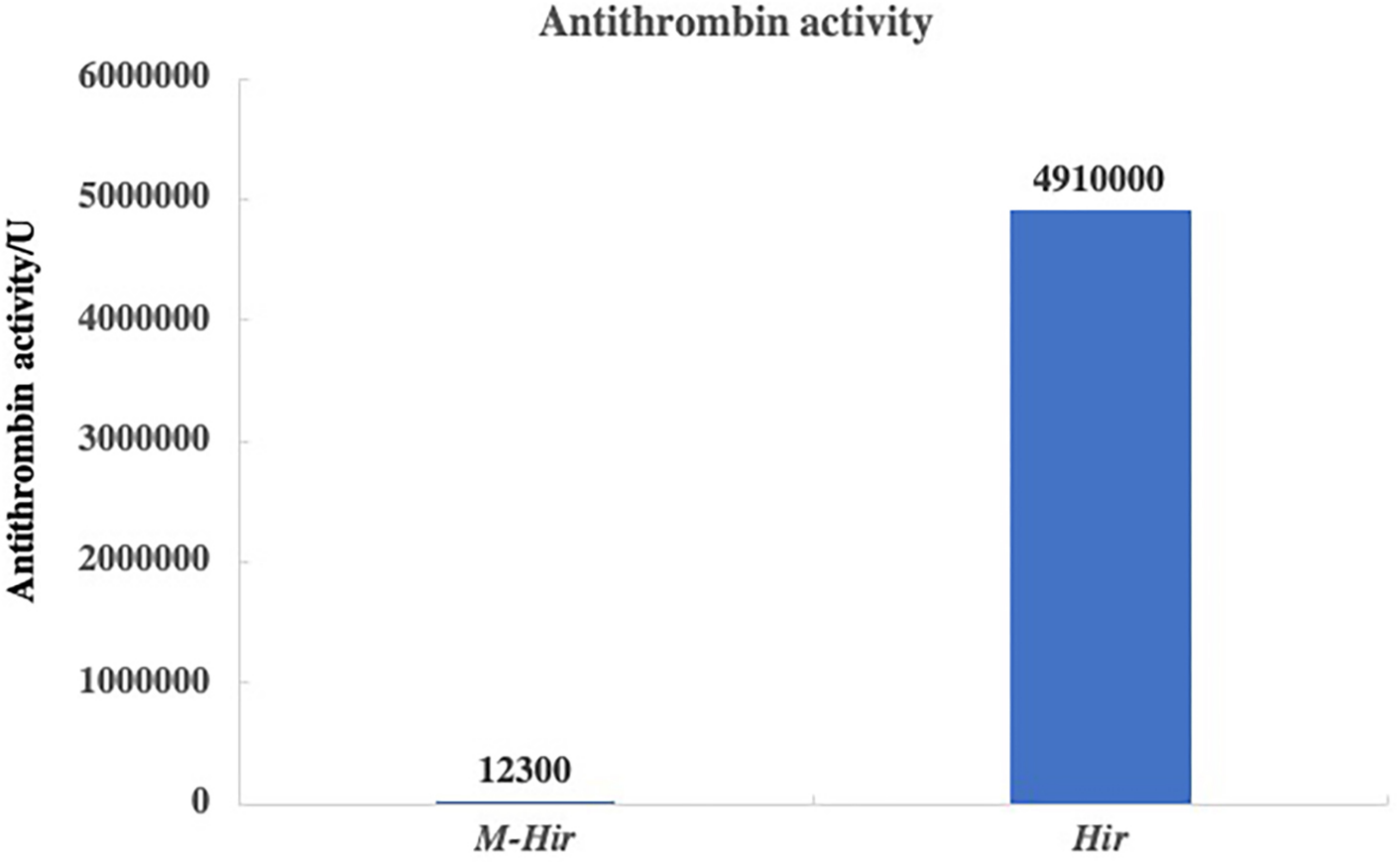
Results of antithrombin activity determination.

**Figure 6:**
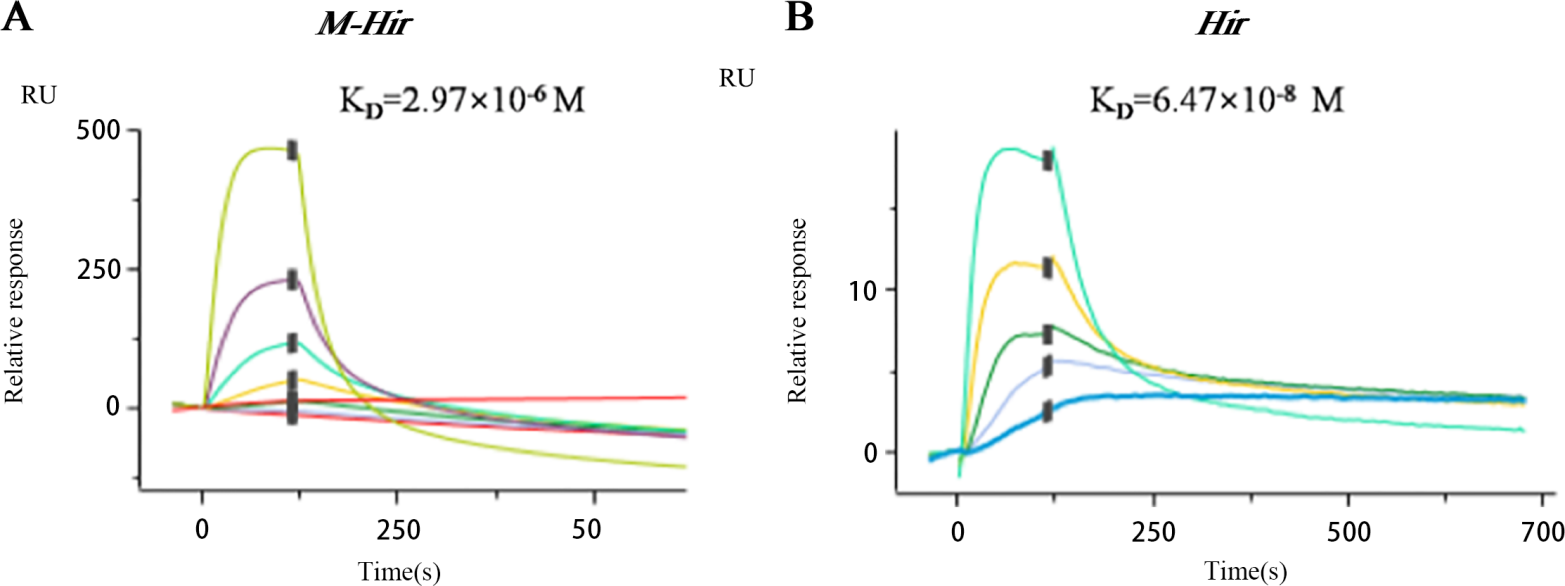
SPR binding sensorgram of the interactions of *M-Hir* and *Hir* with thrombin. BIAcore analysis showed binding affinity of *M-Hir* and *hir* with thrombin. K_*D*_ values represents the affinity of *M-Hir* and *Hir* with thrombin.

The concentrations of the purified *M-Hir* and *Hir* were determined using the Bradford method. The concentration of *M-Hir* was 0.254 mg/mL, and the concentration of *Hir* was 0.167 mg/mL. A standard curve of the association between the thrombin gradient (0–0.25 U) and chromogenic substrate S2238 was prepared (*y* = 2.157*x* + 0.027, *R*^2^ = 0.990). The antithrombin activity of *M-Hir* and *Hir* is shown in Fig. 5. The binding affinity of *M-Hir* and *Hir* with thrombin was measured by SPR (Fig. 6). *Hir* exhibited stronger thrombin inhibition activity and higher binding affinity with thrombin than *M-Hir* (Figs. 5 and 6).

### Expression of hirudin-HN mRNA and protein

**Figure 7:**
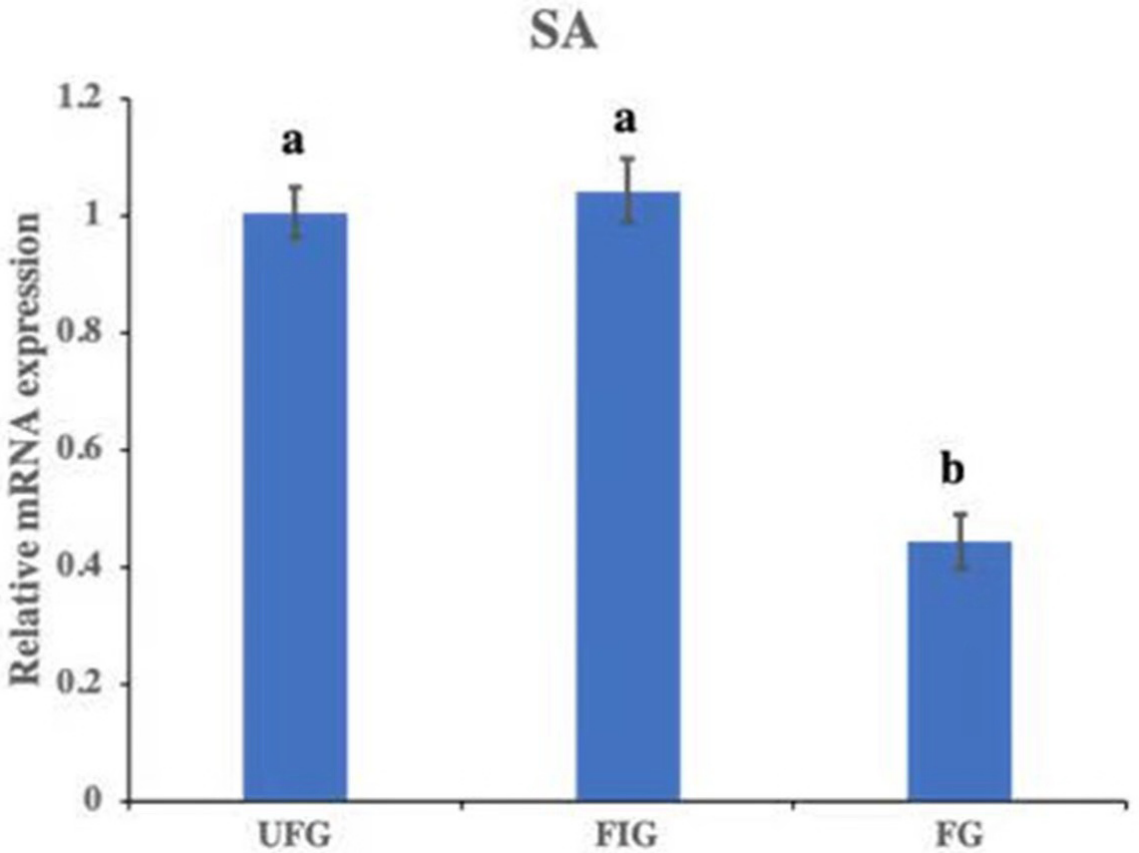
Expression of hirudin-HN mRNA. Histogram showing the relative expression of hirudin-HN mRNA in SA during three blood meal stages. Different letters indicate significant differences (*P* < 0.05).

**Figure 8:**
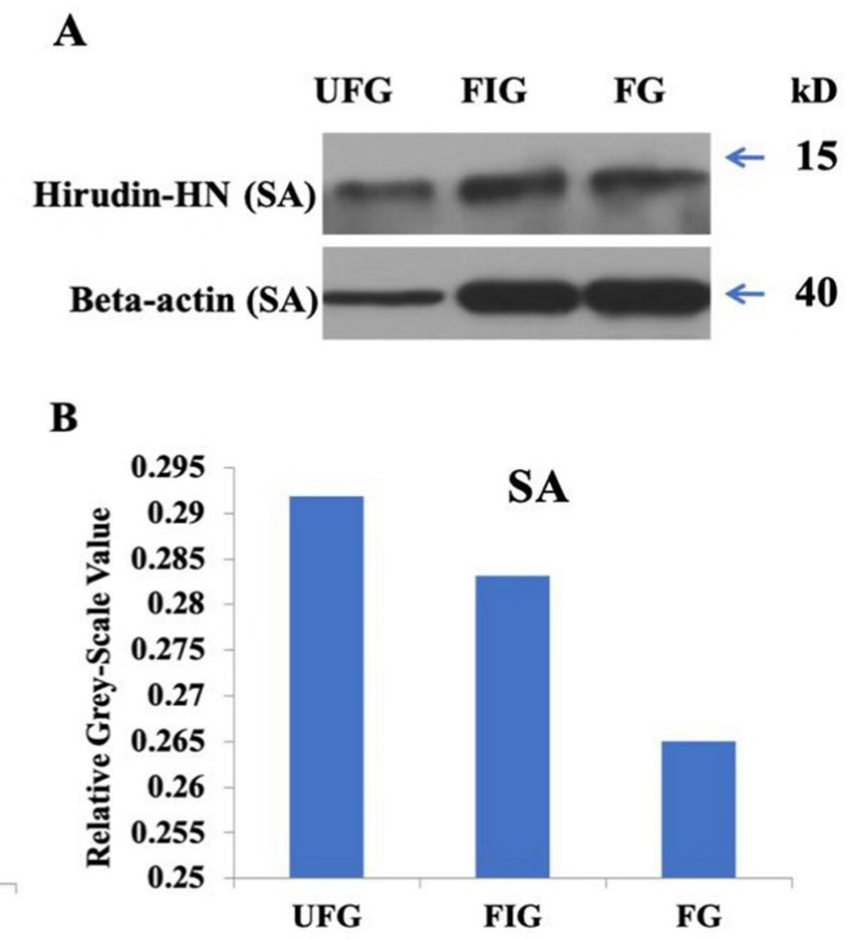
Expression of hirudin-HN detected by western blotting. (A) Images for SA during three blood meal stages. (B) Histogram for SA during three blood meal stages. Quantification of the hirudin-HN protein was performed using ImageJ software.

The relative expression of hirudin-HN mRNA was investigated at three blood meal stages in SA. Hirudin-HN mRNA was expressed in all three feeding stages in SA, and the relative expression in the third period (FG) was slightly lower than that in the other two periods (Fig. 7). At the protein level, we also examined the relative protein content of the hirudin-HN at different stages (Fig. 8A) by western blotting. Hirudin-HN protein content in FG was relatively lower than that in the other blood meal stages (Fig. 8B).

### Molecular modelling of *Hir*

**Figure 9:**
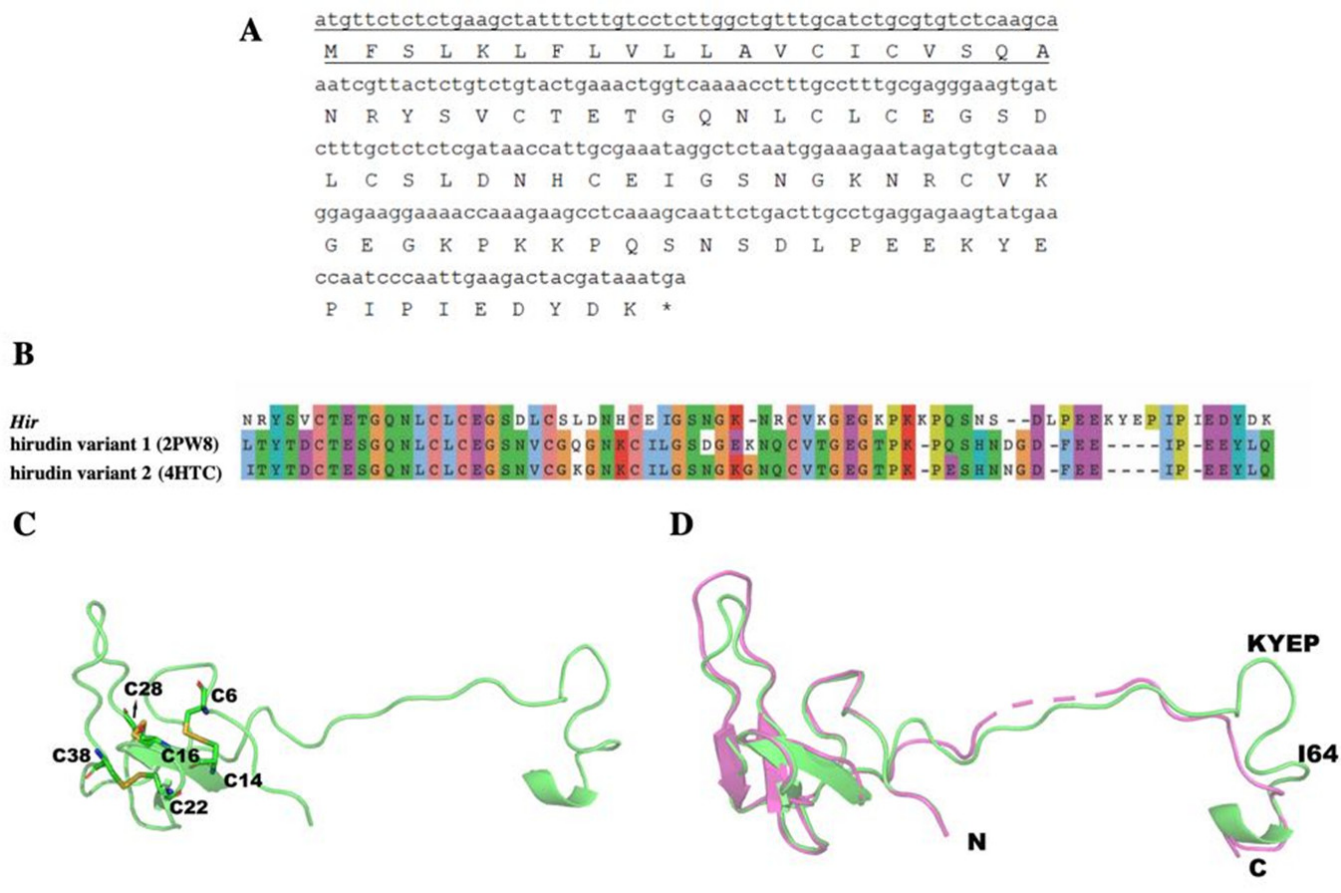
The mature hirudin-HN protein structure. (A) The signal peptide is underlined ; “*” indicates the termination codon, and the other parts are the mature hirudin-HN amino acid sequence. (B) Sequence alignment of the primary structures of *Hir* with hirudin variant 1 (2PW8) and hirudin variant 2 (4HTC). (C) The modelled structure of *Hir*. Three disulfide bonds were marked in yellow. D Structural alignment of *Hir* (green) and hirudin variant-1 (magenta).

The structure of *Hir* contained a typical N-terminal domain, similar to other hirudin structures (Vitali et al., 1992; Liu et al., 2007). There were three disulfide bonds that stabilize the N-terminal compact domain (Fig. 9C). These disulfide bonds (C6–C14, C16–C28, C22–C38) were formed between a β-sheet and three loops, which significantly limited the flexibility of these loops. There was another relatively rigid helix domain formed by C-terminal residues (D66-K69). Between these two domains, there was a long extended loop. As indicated by the sequence alignment, there are two gaps (K35-N36 and S52-D53) and four insertions (K47, L54, I64 and K58-P61) in this long loop (Fig. 9B). Compared with the hirudin variant-1 crystal structure (PDB ID: 2PW8), the long loop (K58-P61) was in the vicinity of the C-terminus (Fig. 9D).

### The structure of *Hir*-thrombin and *M-Hir*-thrombin

**Figure 10:**
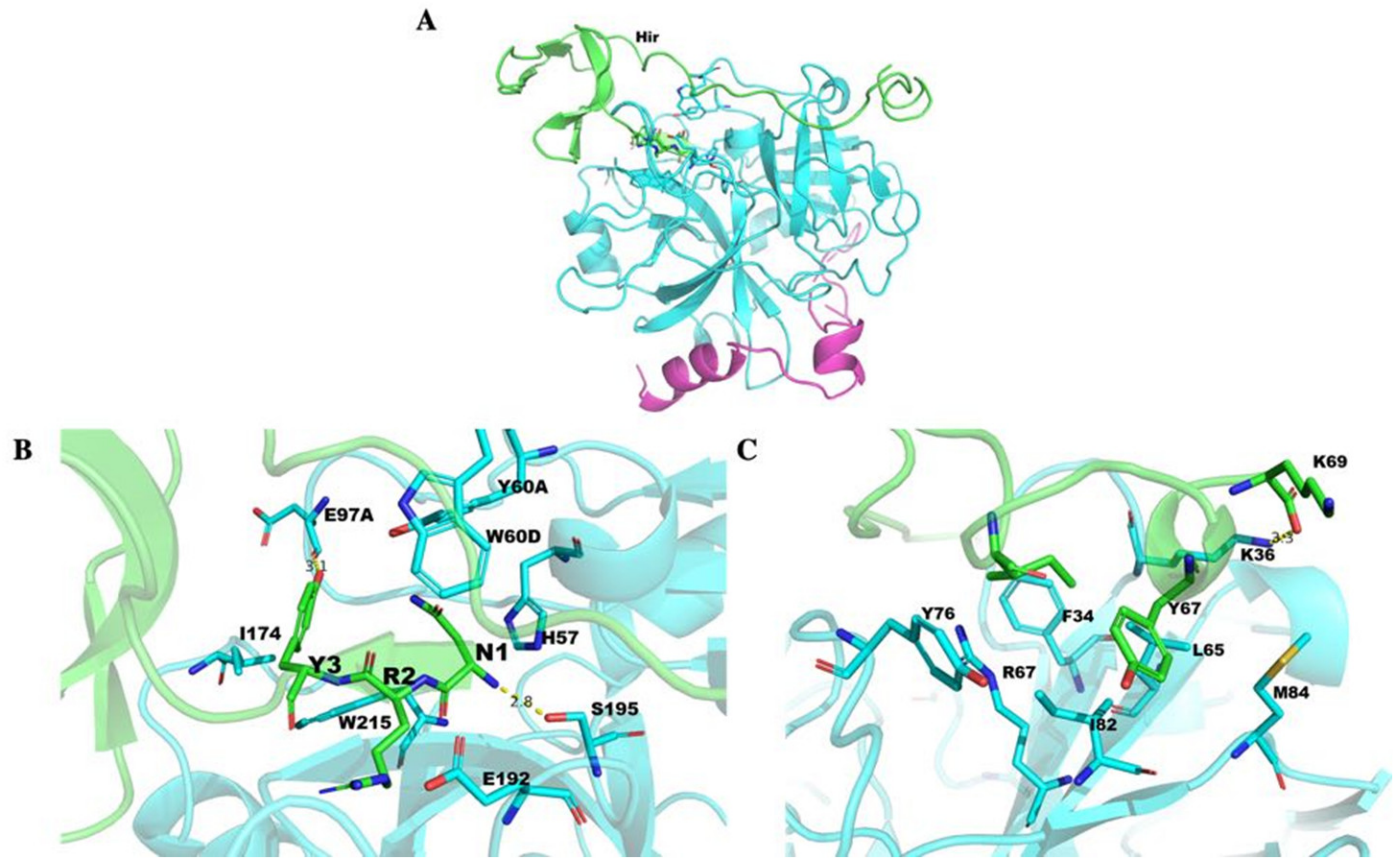
Thrombin docked with *Hir*. (A) The *Hir* (green)-thrombin (cyan and magenta) model. (B) The interaction between N-terminal *Hir* (green) and the thrombin active site. (C) The C-terminal *Hir* (green) binds on the exosite of thrombin (cyan).

The *Hir*-thrombin structure was modelled to explore their interaction (Fig. 10A) based on the crystal structure of thrombin (PDB ID: 1HRT). The three N-terminal residues (NRY) bind to the active site of thrombin (Fig. 10B). This active site is an apolar binding pocket. Two catalytic residues (S195 and H57) are located at the bottom of this site (Sivaraja et al., 2018). There are three polar contacts that form between *Hir* and the active site of thrombin. The backbone of N1 forms a hydrogen bond with S195 of thrombin. The hydroxyl group of Y3 forms another hydrogen bond with the backbone of E97A of thrombin. A salt bridge is formed between R2 of *Hir* and E192 of thrombin. Additionally, Y3 forms hydrophobic interactions with I174 and W215. There are two lysine residues in PKKP, which does not form any specific interaction with thrombin, although they form two strong intramolecular interactions stabilizing hirudin. K47 forms a salt bridge with E8. K46 forms two hydrogen bonds with T7 and T4. The interaction between K46 and T4 is also observed in other variants (PDB ID: 2PW8 and 4HTC). The additional K58-P61 (KYEP) loop is in the solvent environment without any specific interaction with thrombin. The C-terminus binds on the fibrinogen exosite of thrombin (Fig. 10C). This binding pocket is defined by several hydrophobic residues (I82, F34, and L65) and positively charged R67 in a β-sheet flanked by K36, Y76 and M84, etc. There is no specific interaction for Y67 of *Hir*.

The structure of *M-Hir* is similar to that of *Hir* due to their high identity. The N-terminal residue (methionine) of the predicted *M-Hir*-thrombin has a critical clash with H57 of thrombin. This is consistent with its lower affinity to thrombin than *Hir* to thrombin.

## Discussion

The *H. nipponia* leech salivary gland transcriptome was sequenced by the BGIseq-500 platform to obtain 6.5 Gb of data. Analysis and annotation of the transcripts with *E*-values of 1e−5 showed that the salivary gland of the leech *H. nipponia* had a high abundance of transcripts corresponding to proteins that affect haemostasis. In recent years, transcriptome sequencing and annotation analysis have been carried out on the salivary glands of various leeches, such as *Haemadipsa interrupta* (Kvist et al., 2014), *Placobdella ali*, *Placobdella parasitica*, *Placobdella kwetlumye* (Siddall, Brugler & Kvist, 2016), *Pontobdella macrothela* (Kvist et al., 2016), and *Macrobdella decora* (Min, Sarkar & Siddall, 2010), and a large number of similar pharmacologically active substances have also been identified (Min, Sarkar & Siddall, 2010; Kvist et al., 2014; Kvist et al., 2016; Siddall, Brugler & Kvist, 2016). In our study, Unigene5370 and Unigene3091 were annotated as thrombin-inhibiting hirudins; Unigene12407 and Unigene19293 were annotated as an elastase-specific inhibitor, also called guamerin, which was previously identified in the leeches *H. nipponia* (Jung et al., 1995) and *Poecilobdella manillensis*; and saratin, as a pharmacological component associated with the inhibition of platelet aggregation, was identified from the Unigene2825 transcript in *Haementeria officinalis* (2e−42) (Gronwald et al., 2008). In addition, substances that inhibit platelet aggregation were also annotated as disintegrin (Marcinkiewicz, 2005); fibrinolytic activity is a characteristic of destabilase I in *H. medicinalis*, which matched Unigene379 with 3e−55 (Zavalova et al., 1996). The above annotation showed that the *H. nipponia* salivary gland transcripts work together in multiple ways to inhibit blood clotting, which is consistent with the results obtained by transcriptomic analysis of the salivary glands of leeches and some other blood-sucking arthropods, such as *Ixodes scapularis* (Kim et al., 2016).

**Figure 11:**
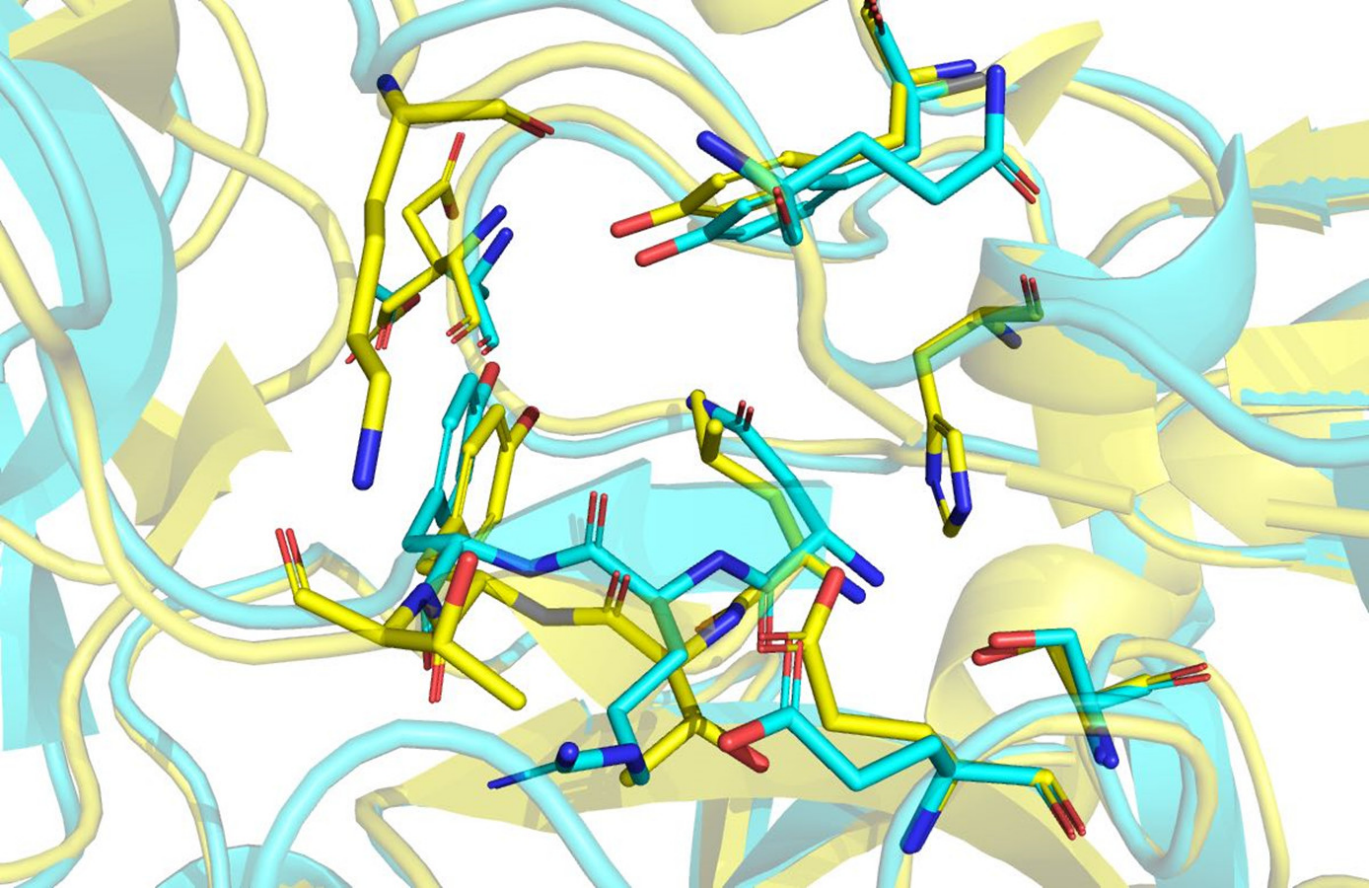
Structural alignment of *Hir*-thrombin (cyan) and the variant-1 hirudin crystal structure (PDB ID: 2PW8; yellow).

Hirudin is an essential pharmacological component of leech-based medicines. Hirudin is currently the most potent natural thrombin inhibitor (Johnson, 1994; Priestle et al., 1993) and interacts with thrombin to form a tight equimolar complex (Szyperski et al., 1992). The mechanism of the interaction between hirudin and its derivatives and thrombin was studied by measuring the three-dimensional structure of the stable complex of hirudin and thrombin (Dodt et al., 1985; Folkers et al., 1989; Grütter et al., 1990; Liu et al., 2007). The results showed that hirudin had a tightly folded N-terminal globular region formed by three disulfide bonds and a flexible C-terminal tail (Vitali et al., 1992). The globular structure at the N-terminus of hirudin occupied the active site of thrombin, and the first three amino acid residues at the N-terminus played a vital role, forming a parallel β-strand to S214 to E217 of thrombin (Rydel et al., 1990). *Hir*-thrombin and *M-Hir*-thrombin structures were modelled to study their interaction with thrombin. *Hir* has a similar binding mode to hirudin variant-1/2. Three conserved disulfide bonds in *Hir* help form the N-terminal compact subdomain. The three N-terminal residues bind to the active site of thrombin. N-1 binds to the subsite defined by H57, Y60A and W60. It is unfavourable due to the apolar properties of the active site compared with those of variant-1 (L1) or variant-2 (I1) (Fig. 11). However, the residue R2 of *Hir* replacing T2 of variant-1/2 should improve its affinity with thrombin with a salt bridge with E192. In addition, in the typical hirudin structure, there is also a tripeptide at the N-terminus (PKP) (Müller et al., 2017) that plays an important role in stabilizing the N-terminal conformation and promoting the activity of hirudin to help insert the N-terminus into the active site (Rydel et al., 1990; Rydel et al., 1991). The sequence alignment shows that there is an additional loop (PKKP) in the long extended loop of *Hir*. These two lysine residues do not form any specific interaction with thrombin while they form intramolecular polar contacts. They should not affect *Hir* binding to thrombin. The solubility of *Hir* may be improved by their hydrophilicity. Additionally, there is a large additional loop (KYEP) near the C-terminus relative to variant-1/2. This loop does not form any specific interaction with thrombin, and it should contribute little to the binding. These residues also may decrease the stability of the whole structure. In addition, the loop may disrupt the spatial relationship between the N-terminus and C-terminus since *Hir* binding to thrombin is limited by the active site and the fibrinogen exosite of thrombin. Overall, the major binding features of the N-terminus and C-terminus of *Hir* are consistent with hirudin variant-1/2 and its high binding affinity. For *M-Hir*-thrombin, the additional methionine residue is detrimental to the affinity due to its potential clash with H57. This is consistent with its low affinity data.

The expression of the hirudin-HN protein and mRNA during different blood meal stage was studied by qPCR and Western blotting. Hirudin-HN expression was concentrated in the salivary glands of *H. nipponia* at both the protein and mRNA levels. There was no difference in the abundances of hirudin-HN between the UFG and FIG periods at the RNA level, but the expression was significantly higher in these periods than in the FG period. However, the hirudin-HN protein was expressed at the highest levels during starvation (at UFG), which may explain why previous studies on hirudin had examined the saliva or salivary glands of starving leeches. The study on the diversity of hirudin in *H. medicinalis* was based on the salivary gland cDNA and genes of leeches (Haycraft, 1883; Baskova et al., 2008; Müller et al., 2016). Most of these studies used leeches that had been starved for a certain period of time (Scacheri et al., 1993). Lemke, Müller & Hildebrandt (2016) reported that feeding-associated salivary protein content was reduced when *Hirudo verbena* started feeding until the fifth day after feeding. For the hirudin-HN in our study, the protein content of FG was lower than that of the other two groups (UFG and FIG), which was consistent with the trend in Lemke, Müller & Hildebrandt’s (2016) research. In addition, similar to the activity in the blood-sucking leech *P. manillensis*, the anticoagulant activity was higher in the head than in other parts of the body (Gou et al., 2016). This result was consistent with the finding that hirudin-HN was mainly expressed in salivary glands in our study.

## Conclusions

The salivary gland transcriptome of the leech *H. nipponia* was sequenced by the BGIseq-500 platform to obtain 6.5 Gb of data. A set of transcripts annotated with anticoagulant activity were screened and included hirudin HV3, guamerin, and saratin. Among these transcripts, Unigene5370 (hirudin-HN) was annotated to hirudin variant HV3 (ALA22935.1) in *H. medicinalis*, and the *e*- value reached 1e−29. Proteins (*Hir* and *M-Hir*) were obtained by prokaryotic expression, and the mature hirudin-HN protein was shown to have anticoagulant activity and thrombin affinity. The N-terminal structure of the mature hirudin-HN protein was shown to be important for anticoagulant activity by comparing the activity and thrombin affinity of *Hir* and *M-Hir*. Hirudin-HN mRNA and mature protein expression were concentrated in the salivary glands of starving *H. nipponia* leeches.

##  Supplemental Information

10.7717/peerj.7716/supp-1 Supplemental Information 1 Raw image for Fig. 4, part 1

10.7717/peerj.7716/supp-2 Supplemental Information 2 Raw image for Fig. 4, part 2

10.7717/peerj.7716/supp-3 Figure S1 The flow chart of the RNA-Seq experimental procedure Total RNA of the three groups was treated with oligo(dT) magnetic beads, and the mRNA was screened for polyA tails. Target RNA fragments were reverse-transcribed into double-stranded cDNA (dscDNA) using N6 random primers. The cDNA synthesized was phosphorylated at the 5’ end, with a sticky ‘A’ at the 3’ end. The samples were ligated to adapters with a sticky ‘T’ followed by amplification. Next, the denatured PCR product was heated, and the single-stranded DNA was circularized using splint oligonucleotides and DNA ligase. The prepared library was sequenced using the BGISEQ-500 platform (BGI) at the Beijing Genomics Institute.

10.7717/peerj.7716/supp-4 Figure S2 Peptide mass fingerprinting of the protein (*M-hir*) The red marker shows the result of matching *M-hir* aa sequences.

10.7717/peerj.7716/supp-5 Figure S3 Peptide mass fingerprinting of the fusion protein (*hir*) The red marker shows the result of matching the fusion protein *hir* aa sequences.

10.7717/peerj.7716/supp-6 Table S1 The assembled sequences of unigenes and of the contigs in Table 2 (transcripts potentially involved in anti-thrombotic activity)

## Additional Information and Declarations

### Data Availability

The following information was supplied regarding data availability: Data are available at the NCBI Sequence Read Archive: SRP151118.
