# Supplementary figures and images for: Identification and characterization of hirudin-HN, a new thrombin inhibitor, from the salivary glands of Hirudo nipponia
Source: PeerJ. 2019 Sep 30;7:e7716. doi: 10.7717/peerj.7716 (PMC6776071; doi:10.7717/peerj.7716)

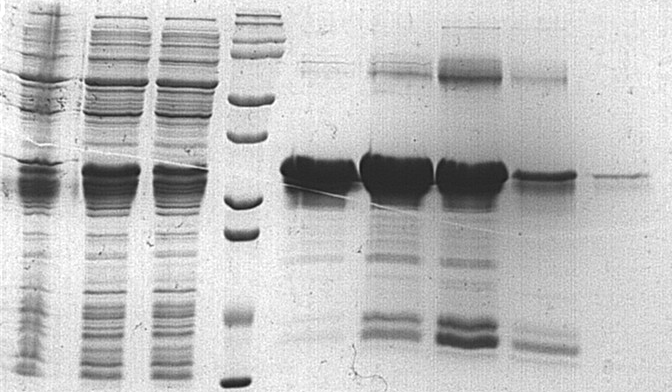

Supplement: Supplemental Information 1 [file peerj-07-7716-s001.png]

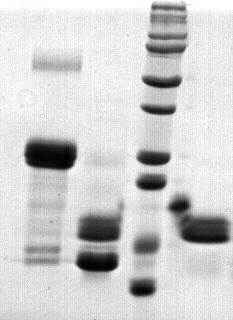

Supplement: Supplemental Information 2 [file peerj-07-7716-s002.jpg]

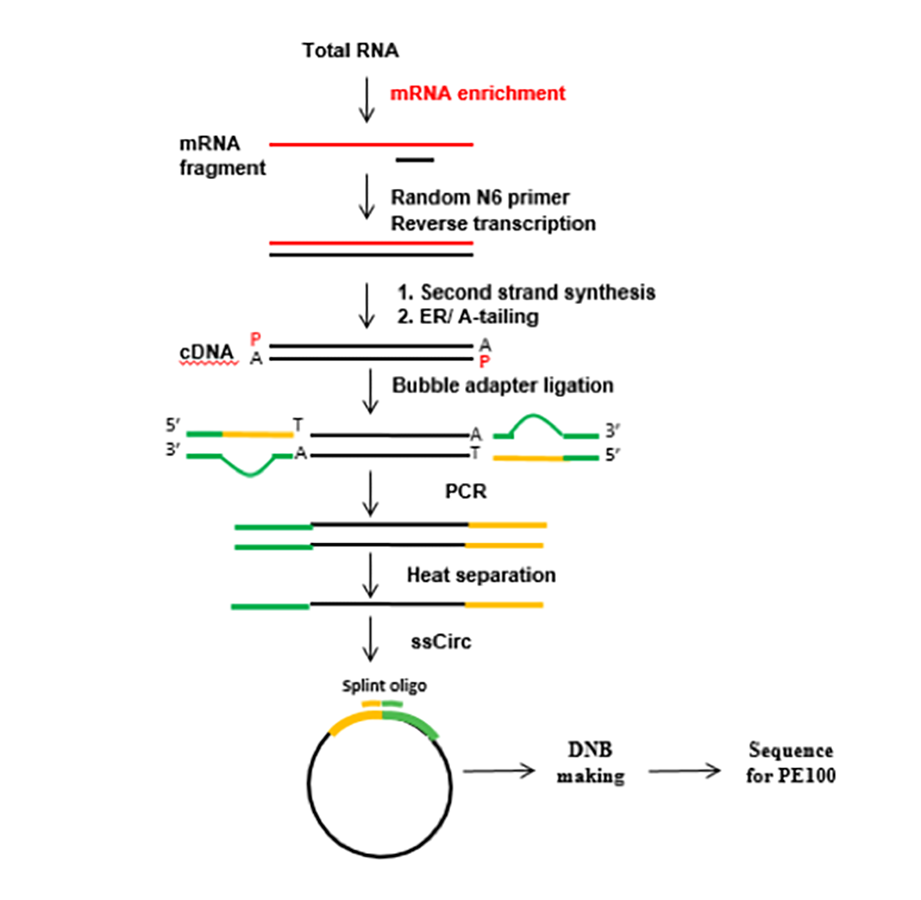

Supplement: Figure S1 — Total RNA of the three groups was treated with oligo(dT) magnetic beads, and the mRNA was screened for polyA tails. Target RNA fragments were reverse-transcribed into double-stranded cDNA (dscDNA) using N6 random primers. The cDNA synthesized was phosphorylated at the 5’ end, with a sticky ‘A’ at the 3’ end. The samples were ligated to adapters with a sticky ‘T’ followed by amplification. Next, the denatured PCR product was heated, and the single-stranded DNA was circularized using splint oligonucleotides and DNA ligase. The prepared library was sequenced using the BGISEQ-500 platform (BGI) at the Beijing Genomics Institute. [file peerj-07-7716-s003.png]

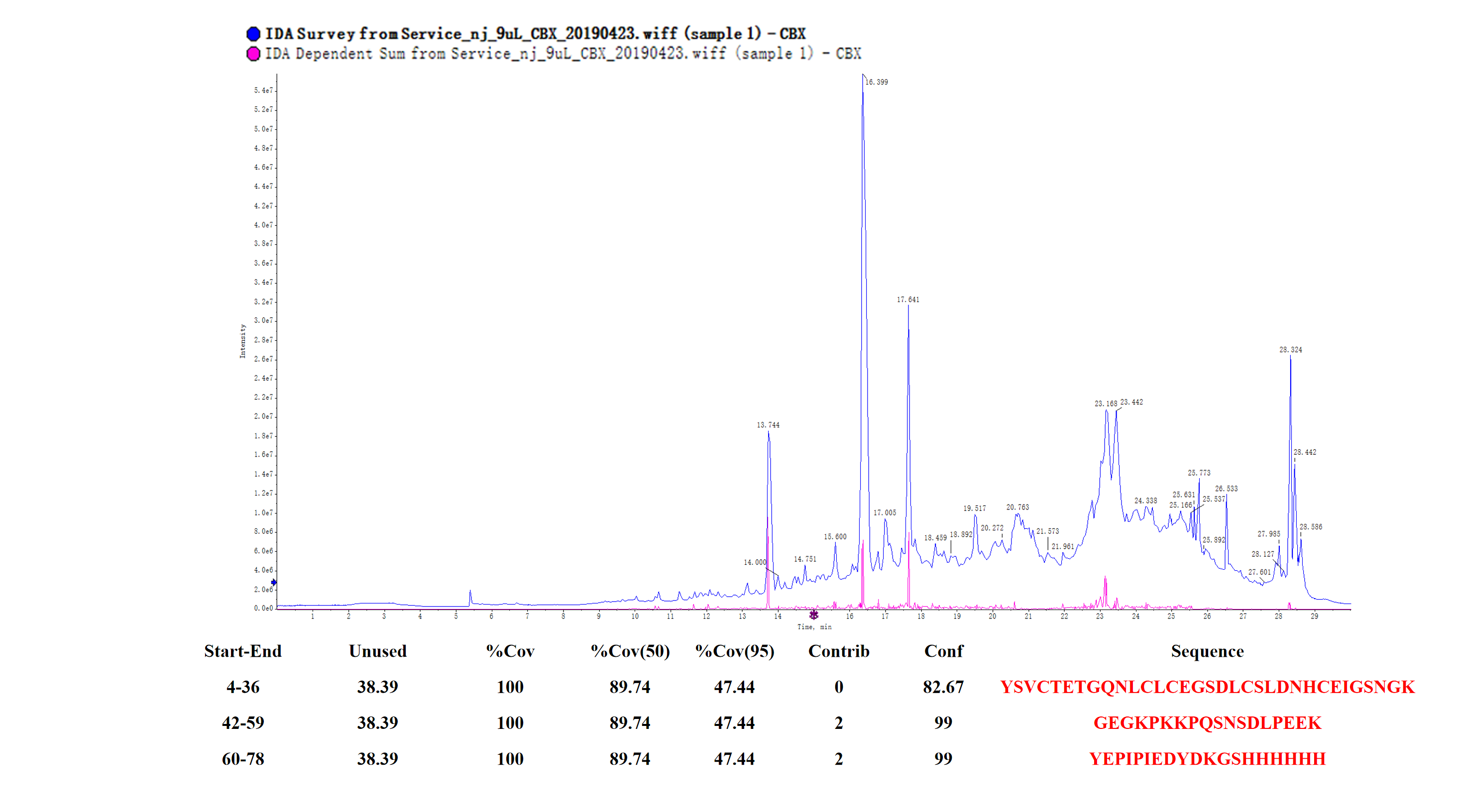

Supplement: Figure S2 — The red marker shows the result of matching M-hir aa sequences. [file peerj-07-7716-s004.png]

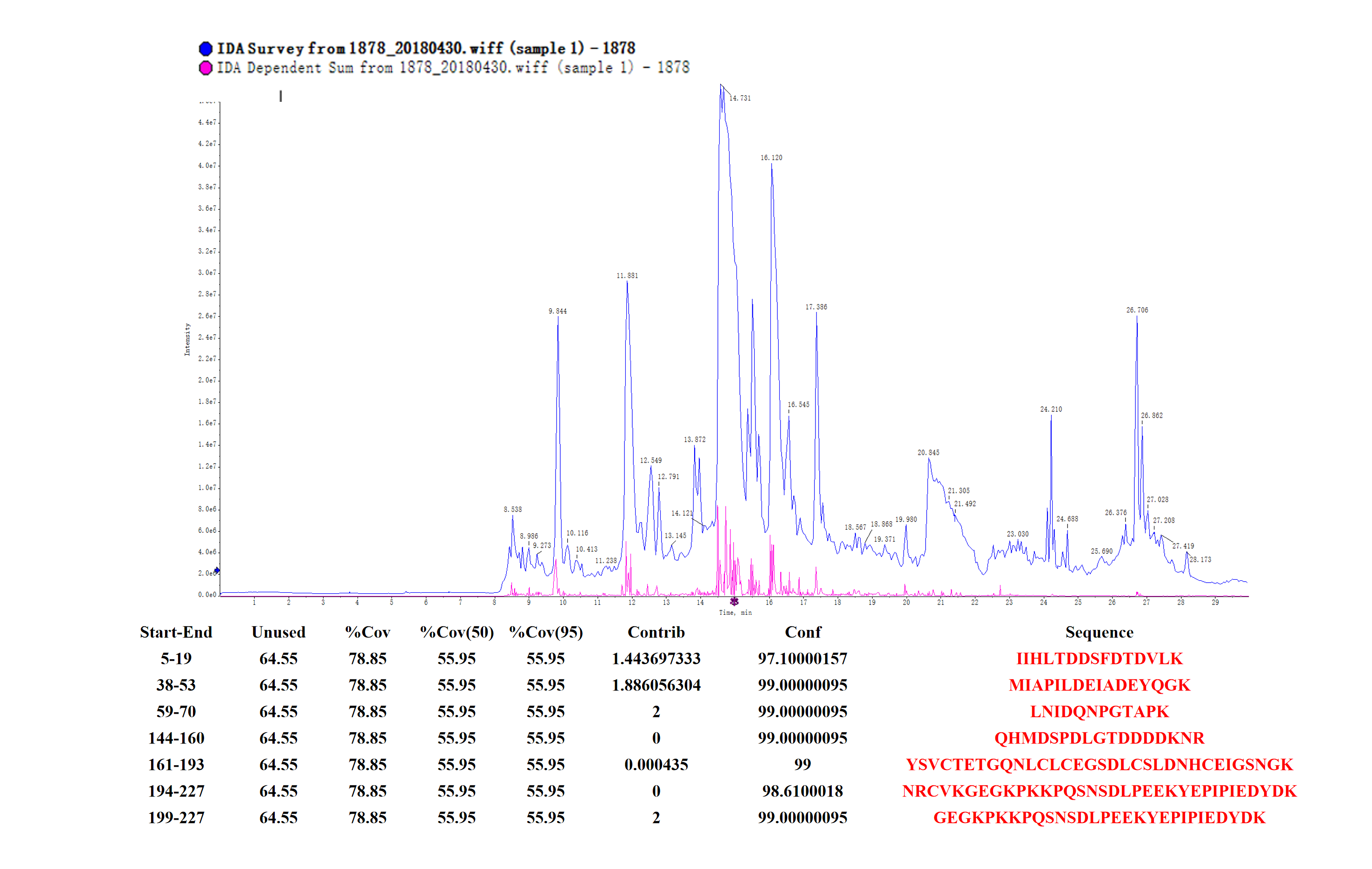

Supplement: Figure S3 — The red marker shows the result of matching the fusion protein hir aa sequences. [file peerj-07-7716-s005.png]
