# Supplementary material for: Identification and characterization of hirudin-HN, a new thrombin inhibitor, from the salivary glands of Hirudo nipponia
Source: PeerJ. 2019 Sep 30;7:e7716. doi: 10.7717/peerj.7716 (PMC6776071; doi:10.7717/peerj.7716)
Supplement: Table S1 [file peerj-07-7716-s006.docx]

Supplemental Table 1 The assembled sequences of unigenes and of the contigs in Table 2 (transcripts potentially involved in anti-coagulation activity)

| Transcript no. | The assembled sequences |
| --- | --- |
| Unigene5370 | ATCATTTCCAGATACATTCGTCTGATCTGAAGATATCTCAAAGATGTTCTCTCTGAAGCTATTTCTTGTCCTCTTGGCTGTTTGCATCTGCGTGTCTCAAGCAAATCGTTACTCTGTCTGTACTGAAACTGGTCAAAACCTTTGCCTTTGCGAGGGAAGTGATCTTTGCTCTCTCGATAACCATTGCGAAATAGGCTCTAATGGAAAGAATAGATGTGTCAAAGGAGAAGGAAAACCAAAGAAGCCTCAAAGCAATTCTGACTTGCCTGAGGAGAAGTATGAACCAATCCCAATTGAAGACTACGATAAATGAAAGCGTATTCTGATCGCAATGAATTTTAAATTAAAAAAACATTCCGTACTTAAACTATTTATCAATAATTAAA |
| Unigene3091 | CAAAGGGATAGAAACAGCTTTTCAGGGACTTCGATCGGATCTGAAAAAAATCTCAGCGATGTTTTCTCTGAAAGTGTTCGTCGTCTTGTTGGCAGTTTGCATCTGCATGTCTCAAGCTCAGCGTTTCAAAGAATGCTCAGAGAGTAATCCAACCCCATGCTTGTGCGAAAATGGTAATCTCTGTACTTCTGGTAACACTTGTGATCTGGGCCCGCCAAAGAAATGCATCGTAAAACAACCTTCCATCTCGGAGAATAAAGAAAGCAAGTCTGATTACGATGAGTATGATTAAACAAACCCAGAATAAGCACCAATTGCAGATAATTTGCTTTTAAAACATTTCCACGGTTGTGATCAATAAACGAAGTTTTCCAGCAAATTATCTGCAATTGGTGC |
| Unigene12407 | TTTAGACAGAGTTTGTTAATGATGAAGACTGCGATCCTTTGTTGCTTTTTCCTCGCTTCACTTCTTACCGTTAGGGGAGTCGACGAAAATGCAGAAGACACACACGGTCTCTGCGGGGAAAAAACCTGCTCCCCAGCACAAGTCTGTCTAAACAATGAATGCGCTTGCACTCAAATCAGATGCATGATCGGCTGTCCTAACGGATTCAAAGTTGATGAAAATGGATGCGAATATCCTTGCACCTGTGCTTAGATTTGAGAGGAAATATTGGAAAAGTTACCGCATTCCTTAATTTCGGTGGAAAGAAAATGAAAATACATTTTCAAATAATAATTTCTTAGTATACTGGTTCTAATGTCATTCAAATAACGTTGGAGAAATATCATTATG |
| Unigene19293 | ATCTAAGGCATGTTGTTGTTGTTGTTCAAAGTTTCTTCGTTGAGCAGAAAATCATGAAAGCTATCGTTCTGTGCTACTTCATCGTTGCTCTTTTGATGGTGTCTTCTCAGGAAGAAGATGGACTTTGTGGGTTAGAAATATGTTCTCCAGCCCAGGTTTGCAGAGAGGATCAGTGCGAGTGCTCACAAGTCCACTGCCGCGTATTGTGTCGATTTGGCTTCAAGAGAGATGAGAATGGATGCGCGTACCCGTGCACTTGCGCAGAAGATGACGGAAGTGTCACACCTTGTGGGGTCTCAAACTGTACTGGAGCACAAGATTGCAGGAATGAAACTTGCGAGTGTCCCTTGATACGTTGCAGAATCAGGTGTCCGTTTGGCTTCAAAGAGGACAAAAATGGATGCGAGTACCCCTGCATTTGCGCGGAAATGCCTAAGCAAGAAGATTGAGGATGTTCCAGTCTTTTGGAAGGAACCCTGAAGAGTTTGGACCACTTCAGAATGTGTGAACATTCAATTTAGAGATTCTAACTCTTGAATTTTTTTCTTTTCCGGTGGAGGGTGATTTAAATAAACCATAAATTTGACGCAAATAAATGTCTTTTTGGCATTAAACTTGACATCGAAAAAATATTTTGTCGCGCAAAACAATTTAAATAAGAATGTTTTGAAGTATATTTACATTCAAATAATCTGAGTAAGAGTTCTCATTTTAACATCAACACACGATGAATGTCATTTCAAACAGGAGCTCAGATGTTTTCACTCCAAAACTTCAGAGTTTTCAAGTCTTAAATATGAACTAAGATATACAATGATATTGAATCAGAATTGCCTATTATTTTTGCCTGGAATAAAAAATACATGGATTTTTAACTTTTGTACATCTTCTGGTGTATTATCAAATTTTTGTGCACTAGTATTTATATTAATTATGATAATTTCATCTCCCCTTGAAAACATTCCTCTTCAGTGAGGGCCTCATCCAACGTTTCAGTGTTGAAATATTTGAACTAAAATCAAATCCAATTTTGTACAAAATGAAAA |
| Unigene2825 | GGAAATTTCAGCAGTTTCCAAACATTTCAACTGCAGGCCGACGATGTTTTCGGATCATATAATAATAACCTAATATCGTAATAAATTAATGCTAAAAATAGCGATAAATACTAGCAAGTGGCCTGCATGATTAATGCTACCGCGCCAATTTACATCTATTGCTATAAAACAAAATATTTTGAATGTTTGCAAACCACGTATAATTTGCTTATATAGTATTAATTTTGACGTGGCGACATTAAACAAATAAAATTAATCTTTTTTCATTTCCGTTCGTAACGCATTATCCACCGACACAGAACCGTGTTTGTTTGTATAGTAACGCATTATCTACAGACGAACTGGCTATAAAGTGACCAATGTCTCAGATGATGTTCAGTTTGAGATTGTTAAATTCAAATTAATTAGTTAAATCATGAAGTATTTCCTGATTTCTTTATTTTGCGTCGCAAGCCTGATGATCTCGACTACTTCTTCAGAGGAACGTGAAGATTGTTGGAGTTTTTACATGAACAGAAAATACACAGAGTTCGATGTCGGTTTTAAGAAATCCAATGATCTTGCCGAATGCCAAAAGACGTGTTTCAACACGGAATATTGCTACATCGTCTTTGAAGACACTGCCAACAACGAGTGTTACTATAATGTGATTGATGGCGAAGAGTTAAAACAAGACAACTTTGTCCAAGATGAAAACTTCAAGGAATATTATTTGCAAGACTGCGATGCAAAGGGTGCAGATGCAGATGCAGGCGACGTGTCCGATGCAGGTGATGAGTCAGAAGGAGCTGGAGAAGACTAGATCATCTATTCTGAGAATTAATGTTTCCGTTACAAATAAATAATTGATCATTAAGTTTCACAAATTATTTTCCAACTGATTAAGCACTAAGTATTAGGATATTTGAAAAAATCCTTCAGTTTATG |
| Unigene379 | CATTCGCACTTTATACGCAATTAGGGCATGATAAGTCTTTATAAAAACGGACCGAGTTGAGGCACAATAGTCTAAGACTGAACTTACAATCTTAATATCTTAATGATGAACTACGCTATCTTTGCTGTCTTAGTGGCACTTTATGTCATCGACATTGCGCAATGCACTGTCCCATCCAACTGCCTGAGATGCATTTGCCAGGTAGAGGGATGCGACAATGAAATTGGAAAGTGCGGCATGGACATGGGAAGTCTGAGTTGTGGTCCTTACCAGATCAAGGAGGTCTACTGGATCGACTGTGGCAGACCAGATGGAGATTACCAGCGATGTGCTAAGAACAAAGCATGTTCTGAAAGGTGCGTCCATGCTTACATGGCCAGATACGCCCTCAGCTGTACGGGTGGACGCCCACCGACCTGCCAAGACTATGCCAAAATCCACAACGGCGGACCGAACGGATGCAACAGTGCAAGTAACCACTACTGGGATAACGTCAATAGATGTTTGGCCTGAAGAGATACGAAGGACCAACATTGCCTCACGTGCGACCGTTTTATAAATGGCTATTGTGATGAACCAAATTCTAAATTAAAATAAAGATATCGTTTATCATCTGATTTTTATTCACAGTA |
| CL1923.Contig5 | GCACTTAATAAATCTGTTAAATAAAAGTACATATAAAATTATGCCACACTTCATATTATATTAATATTTATACGAAATTTATTTCCCAAAACAGTTTTCAAACACTTTATGCTTAAAAAATTTATTTGTTCCTTATACGTTCTTAAAATTGAATTAAAAGGCTTTTAAAGACAAGAGCCGATTAGAGAATTTTAATTCATCTCAAACATTTCTGCACCTTGTTCCAGTAACCAGCAGTGGCAGGACTCTTACAACCACTTGGTCCACCGTTATGAATCCTAGCGTAGTCCTGGCAGCTTGGAGTTCGTCCTCCTGTGCAGTAGGTTCCATACCTCTTCATGTAAGCTCTCACGCAAGTCTCTGAACAGGCTTTAATTTTTGTGCAGGTTTCATAGTCTCCCCCTGGTTTTCCACAATCAATCCAGTATGGTTTCTTAATCTGGTATGGTCCGCAACTCAGGCTTCCAACATCCATTCGACATTTTCCAATTTGATTGTCACATCCTTCAACCTGGCATATACACCTAAGGCAAGAATCAGTGAATTGGCTGTTCACCTCGCCAGCGGCGATTAGAAGAAGAGCGAGACAGATACAACTTGAAGTTTTCATCCTTGTCCAAAAGGTAATTTTTCATTGATTCAACTATTCAGCTATTTATTTAGCCAAAATTTGAATGGTACTCCAGTAAGTCCTCTAAAACTTGAAATAGTGTTGATTCTCAGCATCGTAAATATTTAAACAAATTCTATCTTGAGACATTAAATGATATCCACTATAAAGACATTTTGAT |
| CL359.Contig2 | ACAGATCTCTGAGAAGAAGAAGGTCTTCGAATAAGAATTTGATAATAGATTTGTTGATTATAATCGACAACTCCATGTACACGAGATGGCTTAAATGGAGTAGTCATGACAGGCCTGCATGCATTGAGCAAATTCAGCACTATTTGGCTTACATCTTTGAGGAGGTTAATAAAATTTATTCTTCATTGCCCTCATATCAGATAACTTTGAATGTCGTCCAGATATTTATAGCTGAGACATCATCACGATCAAACTATCTCGAAAATTCTATAGCAAAACAATATGAAGACAAAACGAAGATAAATTTTGAGGCAGCTTTACCAGGTTTCTCTGAATGGGTGAACAAACATGAGATTGGAAAGGGAAAAGCAAGCACCGAGAGGTTTTTGAAACAACATGACCTTGCAATCGCTTTTACTGACTATAGCGAAGATCATGTAGGCGGAGCAGCTGGCTTGGGCGTAATATGTTCCAACTATTCACAATCGATTGTGCATGCAACAGACCTAAGCTTAACCATCACAGCAACGGCCCATGAAATTGGGCATGCGCTCGGAAGCAGAGACGATGGAGATGGTAACCAATGCGACTCCGGCTATCTTATGTCGAGGGCTTCGACAGATAAAGATATCACAAATTGGAACAAATTTTCTACTTGTTCTATCGAACAGATAAAAAACAAAATTAAAGCATTGAAAAGATCTGGACAAAACTGTTTGGATCCATCTAAAACGCAAAGGGAACATTTTGAATTCGGAACATCTTCTAATCTCGGTGTCCATTTAGGAGCAGATATGCAGTGCGGATTAAGATATGGAAGGGGTTATAAATTCTGCTCGCAAGGATTAAACACCGGTTCGACCATGTGCAATAAAATGATGTGTCTCCATATGGAAAACAAAACTTGCCACGGAAATGGGACATTTCAAACTGCCCTCGACGGCACAACTTGTGGCAAAGGAAAATGGTGCGTGAAAGGGGAATGCCTTCCCGGTGCTCCTGTTAAACCTGAAGATAAAGATAAAAAAGATACATGTTATTTTGGAGATAAATCAGACAAGTTCAGCATCGACGAGAAAACGATGCAATGCGAGGACCTCCTTAAAAAACCCAAATTGTGCAAAACCGCGGCAGGATGGTGCTGTGAAACCTGCGAAAAATTAAAACATGAATTAAGTGACACGTGCGATGACGAGGGGCAGTTTAATTACGGTGGGAAAACAATTACTTGTGAACACATTCTGAATAATCCATGGTCATGTTATGATCTGGACCATTACTGCTGCAAAGCATGCACAGATTTAAAAAATGAAAAAAAGAGTTCTGACGCTAACTGTGCTTATGGAAATAGAAATATGAGAATACAAACGGATAGAGGAATAGTTGGATGTGAATTTGTTCGAGAAAGGACCGATCTCTGCGACCTAAACGATTATAAAGTAGGGTGCTGTGAAAGTTGCGACGAAGTAAAGAAGGCACAAAAGACACCTCCGATATCAACAGAGGTTACGCAAAGCACGTGCGTTGACGAGAAAGGGAGGTTTGACCATGCAGGAAAAAGGATTAATTGCAAACACCTTCTGAGCAATGCAAGCCTATGCTATGAATTTGACAAACGATGTTGCAAAACATGCGCTGACATAAAAAAGAAAAAAGCTCTGCCTTACGATAACTGTGCATATGGAGACCGGTACTCCGTGATTATCGTGATGTTTGGGAAAAAATACGGCTGTGAAAGTGTCTTGTCGAAACCCACCGGTTGTTCACCTGTTGACATTGAAAGATGCTGTGAAACGTGTGGAAAAGCTAAAAAGAATGGTCTTACTCAAACAACAACAAAAAAGATAGAAGGGACCACTTTGGACTCGTGCGTTGACACTAATAATGTTCGAGGAAAAATAATAGACTGCAAATCCTTGCTGCAAACTCCAGGAAAATGTTATTCGCTAAAACAGTATTGCTGTAAAACATGCTCCGACATAAAACGCCGTAAAGTTGCCCAATTAGGGCCAAGGTGCCCATACGGGGATATCTGGGGTATGAAGGTGACAATTCAAAACATCAAATACGAATGCTCGGCTGCGAGTTGGAAAGATGGCATATGTTCGAATTCTTTCAACAAACAGTCCTGCTGTGAATCTTGTTCCGGCGTGAACTTGGGACAGAGGCCCATCGTCCCTGAAATTAAATGAGAAAGACACGTCTAAAACAAGTACGGATCGTCCTTGAACAAAGCAATTATGAAATATATTTAAAATGGAATTACAACTCAATTAACACGGATTTTGTTGAATTATTTTATAATAAAAAGTAAACGGTTAT |
| CL2866.Contig1 | TCTTTATTTTGCGATCAATTTATTTAATAAATGAATTTTCACTCATAAATCAGACAATTTCCAGTTAAATTTATGTCTTGAATTTCTTCTTTTTCATCATCCAGTATTTTATCCCGATAAGGCTTTTCTGACTTTATCGCAGCTTTCACAACAGTTTTTATTAATACCGAGGTCATTACAAGCACTGTATTCTTGGAAGACCAATTCACAGCCATAAATTCTGCCACCCATTCTTATCTGCATTCCTGCGTCTCCATATGGACAGTTTGGTGAGCCAGTCTTTTTAACTTCTTCGCAAGATTGACAGCACCATTTTCGTATTCCCGGAGTGCCGCACGAAGTTGGCTTTTTTTTTATATCTTTGCATGTCATTAAGTTTCCGTTAAATCTGAACATATCGCTTTTATCTCCGAATATGCAAGTGTCTTTACCAGCTTGTCTGTTCGGATTACCATTAACGCATTGACCTTTTATGCACCATTTACCACTGCCGCAAGTTGTGCCATCCATGGCTATTGGATATCCGCCATAGCACATATTGTCTTTGGTGTTGAGACATCTCATTTGATTGCATACCGTCAAATGAGTGTCCTCCCCTTCCGAGCAGAATTTATGATCACTTCCGAATCTTAATTGGCATTGTTCATCGACACCGAAGCGAGTGCCTGGACGAGAATCGAGTAGTTGGTCGAGATTAATTCTTTTACTGGATCTTGACGGATCCATGCAATTTTGGTTTGTCATTTGCAATTGCACAGCCTTTGCTACAATTTGTTTCATCGTGCATGTTGACATCGTGTCTCTGTTCTCGATCTTTGCATTGGTCATTATGGGTGACATGAGATAATGGGCATTTGGATCACACTCGTTGTCTGAGCCATCGTGTTTGCTTCCAAGTATGTGTGCAAGTTCATGAGCTGCTGTCGTGATGGTATCGTATTGTCCTTTTTCGTGAATGAGTCCCTGTGCATGATCTCCGCAGACAGTTCCGAGGTAAGCCAATCCTTCGGTAGGACTTTCGCTGTAGTCGGTAAATACAACTGCAGCATCATGTTCCGGTATAAACTTGTCCCTATTTTGTACCTCTCCCCTCTTAGGCGTAAATTTCAACACCCATTCTCGGAAAGCTTTTAAAGCGTTCTCAAAATCTGCTTTTCTGGGGTCCAAATGATTTTTTATACGTTTGTCTTCAAAGTACGAAGACTGTTGAGCTGTCCCTGCAATGAAAATTTCTACGACAGTGACGAACAGTTTCGGTGAGGCCATTGTTGCGTAAACATTATTTACCCCGTTCAAAATATGAGCTAGATAGTATCGCATTCGTTCAACCGTTTTGGTTTTGTCGTTGTTGTTCATTTTAAGCCACCTGCTATACATGGCATAATCAATTACAACCATCAGATCAACATGCAGAGTAAGTTGTGCATTTCTCTTTCTTCTTTGAGATCTTGTCTCGGTTGAATTATTTCCATAGATTGCATTATTTGGCCCTTTCATCGGATTAAGAGTTACTTCTACAGGCTTTGGAGTGGTCACGTTAAGTTGCTCAACTGTTACTTTGTATACAACGCTCGGAGTGGTCTGTTCAACGAACTTAGTATTTGATTCTTCTACATCGTCGTCAACAGGATTGTCTTGGGATTTGTCTACTTTGTCGATAAAAACAACGTATTTAGTTCCGTTCAACGTGAATTCGTGAACAACATCGCTTGTTTTGTTGGACATTTCATGGTAGTTTTTTGGTGTCAAGCTGTATTTTATTTCTTTGTGGACAAAAAACCCGTAGTATTCTGCAATAGTTGCTCCATTTTCATTCTTCATGACCACACTGAAAGATGCATCTTTTGAGTTGTCGTTGTAAACGCTAAGCACTTCATCCTTTTTGTGGTGCCACACTGTAACATTATTATTATCTCCTCCAATAACGACAGGAATTTCCTCAAAGTTATCTCTTTGGTTCTTCACCAAGTTTAGTGTAACAGTCTCTTTGTCTATTGTTAGGCTGAGTACTACTTTATCAGGCAAAGAAGGGTGCTTGTCACGACTGTCGTCTGTTCCGTTTGTCCAACTCACTTGAACATCTTCAGATACGACAGTTTCTGCCCTGCCATGAACAATGAGTCTCGAGAGTATAATAAACCAGATTAAACCCATTTTCATCCAGTGATTCGTCTTGGG |
| Unigene17477 | AAACATTTTTTTAACATTTTATTATAGGCAACAAATTTTGGGTTCTTTTTGCATTAAAAATCCTTCGTTAAACTGAAATTTATTTATTACGGCAGGTGTAGCAGCATTGTTCCTTGCTTGATTTACAGTGATAAGCAACACAACCAGGTGACTTATCGCCATATTCGCAACCTTTTGGAGCGCCCGCTTTCTTGAGAGATTGACACTTTTTGCAGCAGTTGTCTTGAGTATCTCCTACATAACATTGATAACCAGAAATAGTAGAACAATAACTAGGGTCCCAATCAAGACATTGAGGAGTTTTTGAAACTTTGCATGAAGAACAACACTCGTTTTGGACCGAAGTTTGTTTACAAATGACAGGTTGTTTCTTGACATCGCTTTCACAAGAATCTCTTTTATCTCCGTATCGGCAGTCTTTCTTAGTTTTATCCTCAAAATTTTTACAAGTCTTGCAACAAGTATCTTCATTGGCGTAACATTGATAACGTTGCAACGATTTACACCAGGAAGCTTTATCACCAAATTGACAATTATCTGAGACATTAGCTTCACAAGTTCTGCAGCATGTTTGCATTTTGGCGTACGAACACTCAGACTTTTTGCAGCCATAATCTTTATCTCCATATTCACAACCAGTCTTTTCAGGTTTGTAAAATTTACTACAAGTTGCACAACATGCCAAAGCAATGTCAGGCATGTAACATAGAAAAGATTTTTTGAGTCCAACAGTTTTGCAGGATATCTCATTGCTATCTTCGTCCAGTACATATCCCTTCCAGTCACCATAAAAGCAGTTTTCTTGTCCAGAGCGAACGTTGCGGTCAGGTGAACAGGTTTCGTCTTGACACGATCTTCCGCTTGCACATGGCGTTCTGTCCGCTGGGGCTGCCTTGGGACTTCTTTCACAAATTTGTCTGGTATCGTTCCAGCAAGTCATTTGTCTGCACATCGTTTTGAAGTCATCACTACCACCCGCGCAAAAGAAGGACTTTGGTCCATGCAAGATCTGACACTGTGCGTCGGCATCGATCTGAGTTCCGAGAGGAACTTTTGTGAGTTCATCCAGATTAATGTATTGTTTCGCACTTCGAATCACCAGACAGTTTAAAGATTCATTATTCATTTTCTGGATATATTTTTCAAAATACCGAAGCGAACACGGAGAGAATGTATGACGATATTTAAAGTTCTCGTCAGTGATGTATTCAACCCCTGCTGCCATTATGTACTGGTTATTGTAAGGACAATCGCTAGAATTATCGTGATGAGATCCGAGATTATGCCCAATTTCATGAGAAGCCGCATGATAGGTATCGTAAAATCCCAATTCCCTTGTCACAGAAACTCCGTTTTCTGTACATGCTGCCATAGGCCATGCCAATCCGTCATAGATTCCCGTGTCATACTTGGTGAAAAATGCAAAAAGATCATATCTTGGAAAGCGTTTCTTCTCATTTTTTAGCCAAGATGTAAGATTGCGAAGTACAGTGTCGATATTCACTTCATTTTGGGAGACTTTGTATTCATTTTCAACAAAAGGGACTTCACGTTTTGACTTTGCAATCATTATGTCAACAACTTCGAGGTCAAGTTTTACCCCTCCAATTGTTACAGGATTGTATCTCTTCTTGATCTTTTTGAATATGTGAGCGTAAAATGTTTTTAAATCTGATATGGTCTTGTCTGATTTCCCTTTGTTCAATGTAAGCCACCAGTCATATACAGAATAATCAAGCATGACCAGAATTTCCATGACATAGGTTTTCGCTCGATTGTTTTTTATTATTTCATTAGATTGTATAGATTCTTCATCGGACATAAGTTTCCAGCTGCTTGTAAACGTAAGTTCGTCGTAGATTTTGCTTTTCTTTTTATTTCCGATTTCAACTTCTGCATTGCTCAACGGCAGCAAAAGGAAAACTACTAAGAGAATCATGTCAGAATTATAACATAGTTCTA |
| CL2798.Contig2 | GTCGGTTCTCCAACATGGGAATCTTTACCTGCTTCATATTGGTGTTATCAATTGTTCATGGAAGAACGGACGCTGCTGTCGATGAAGCCCAGGTGAATTGGACCAATAGTACAAGCCCTTCATCCGGACAAGACAGTCATCCTTCTTTGCCTGATAATGTGGCGCTCACTCTGACAATTGGCAACGAGACTTTTGAACTGAATTTGAACAAAAAGCAAGATGATGACTTTGAAGAGATTCCAGTTATCGTTGGAAACAATAAAAATGTTAAAACTTGGCATCACAGCAGAGATGAAGAATTTAGCGTCTACAATGATGAATCGAAGGATGCCACGTTCAGTGTGGTTGTGAAGACTGAAAATGGAACAGTCGTTACGGAATATTACGGGTCATTCGTTCAAAAAGGCATTAAATACAACATGATGCCAGAAAACAATCAAGATAAAACAAACAAATTGATCACTATTACCCACAAGTTTTTACTGAACGGAACACGTTATGTGGTTTATGAAGACATAGACGAAGCAGAAGGCAAGGAACAGGACATACCCGTAAACGACGTATTTGAAGAAACCAACTTAGATGAGGAAGACAATACTTACGTTAAAGACAGCCAAAAATCTGCAGATTTGTTTAAAGGTGAAAGCGTAAATAAAATTTCAGCGGACAATTTCAACCTCCCAAGATCTTCAAGAAGCAAAAGGGAGGTATCAATGGATTTGGTAGTCGATCTGATGGCTGTTATTGACAACTCCTTATATAGAAGGTGGCTTACACTAAACAGTTACGACAAGGACCAAACTATCGAAAGAATGAGATATTATTTGGCCTACATAATTAACGGGGTAAACAATGTTTACAAGACAATGGTATCACATCAGTTATCTGTCAGCGTAGTTGGAATTTTCATAGCCGAGACACCAGAACAATCCGAGTACTTTGAGAATCCTTCAGTAAAAGTTTACGCGGACAGAAGAGCGGCTAGATTAGAAGAGGCTCTGAATGGCTTCAGTAAGTGGATAAAGAACCACACACTCCAAAATGGAGAATTGGCAACAGACGAGAAATTTTTACCTGATCACGACGTTGCAGCTGTTTTTACTGACTATCGTGAGGGTAGCGTAGAAGGACTAGCTCCTCTGGGCTCTATCTGTGGAAAGAAACCACAAACAGTGGTTCACGCGACAGGACAGTACGATACCATATCAAGAACTGCTCATGAAATTGGTCACATTCTTGGCAGTGATCATGACGGAAATGGTAATGATTGCAATCCAGCTGATCACTACCTCATGTCACCAATATTGACCCACAGAAAGATCGAGAACCGAAACAATTTTTCCAATTGTTCTATAGCTCAGATTGTAAACAAAGTCCTAAATTTGGAGAGGAAAGGAAAAAACTGTCTGGATTCGTCCAAAAAACAAAGAGAGAAAATAGGTCTCGACAAATTACTGAATACTCATCCAGGTTTCAAATTTGGACCAGATGAGCAATGCCAGTTGAAATTCGGCAATGGATACAAATTCTGTTCGAGAGGCATTGATACTCACTTGACCATGTGCAACAGAATGAGTTGTCTCAAAATCGGTGACAATTACTGTTACATAGGACATCCACTTGCCCTCGATGGCACAACTTGCGGAAAGGGAAAATGGTGTATTAATGGGAAATGTGTTTTTGGTGCCCCGGTAAAGCCAGGAGAAAAGGAAACATGTTACTTTGGAGACAAATCAGACATTTTTACGCTCGGTAAAAAGTCAAAACGTTGCAACGATTTTCTAGATGAGCCTAAGCTGTGCTACAAGTATGACAGTTGGTGTTGTGAAACCTGTGCAAAAATCAAAGAGCAAAAAAACATTCCTCTCGCAAATTGTGCTTATGGAGACAAGGGTATCGACTTCATCGTGGATGGCAATGTTCATGGATGCGAGCTGGTCTTACAACGTCCGTACTTGTGTACCGATCCAGAAAACGCTGATTATTGTTGTGAAAGTTGCAATATTGTGAAGGATTCTCATTTAGCAACTGGGGAAGAAGAATGTGAAGATCAAGAAAATTACAAACAGCAATGCAAATCTTTGCTAATTTTTCCCAGAAAATGTTACAAGCTTCAAGACTATTGCTGCCAAACATGTGCCAAAATTAGACTTGAAAAAGTTGCCGAACTTGGACCAAACTGTCCTTACGGAGATCAGGATGTACAATTGGAAATTCGAAATAGACGTTACAGCTGCTCGATAGTGCTTTGGAATCCAGATATCTGTACTAACTCACAAGTTGCCCATTTGTGTTGCGAATCTTGCTTCTATGTCAAAGAAAACAATTATTAGTGAACTGCACTGAAAAATTCAAGTCAAAGATATCCTGGAGCGAAACATGAATCGACTTTATCAAAGAATTTAATATACAATTTGAATTTAAATATTGAAGTCAATTAACAAAAATATTGTTGAA |
| Unigene25705 | CAACGGACGAAGAACACAACCCTTTATAAAGACTCCTTGTTAGACTGAAAAGTTGTTAACCGGAAAATCGGTCGGAATCGTTATGAAGAGCTTCGTTTGTGCTGCTTTGGCTGCTCTCGTCTTGATCAAACTCGGACATATCGGGGTTCGAGGTGAAGAAGTAAGGGTTATCAATGCTCTGGTCATTGTGGACAATTCTCTGTACACATTAAAATGGAAAGACTCAACCAATGATGAAATTGAATGTTATTTCAACACGATCATCAATGGTACAGCCAAGAGACTCGAGAGCATTGGAAATCCTGCTATAACCGTTTCAATGTTTGATATGTACGTCATCAAGGATACACGTGAGGCTGATTTCGTAAAATATTTTAATTATTTCTATGGAAGCACCGCCCCTAGTATCAACCCCAGAATTACAAGGAAGGAATTCTATAACTGGTTGAACCAGCCTGGTAATGAATATTCGTCATACGACTATGACGTTGTTATACTGTACACGAGTTCTCAGGTTGGCACGATCAAGGTATCTGCCCCATTGGGCAGCCTCTGTGGAGATCAACCACCAGTGATATTGGTAACTGATGAGGGTCTTTTTAATTCAATCGATGCCACTGCCCATGGACTTGGATTGAGCCTCGGATCCGAAAGAGATGGCGATCACAACAAATGCGACATAAACGACCATTACATCATGACAACAAGAAGAAATCAGATGGAAGGAGAAGTTTATAAAAATCATCTCACATTTTCAAAGTGCTCTATTGATTACATCAGAAGTTTCCTTAAAAAAAAAGGACCGAAATGTTTGGCAAAGGGGTACTTGGGCCAATGCATCGATGAAGAAAACTGTGGCCTCCAAGATGCTGGGCAGAGGTACCCACTTGATAAACAGTGCCAATTTGCCATTGGAGCTGGCTCAGAACTGTGCATTGGTAATTTCAAGGTCGATAGGGAGATGTGCACGGAGGTATACTGCAGAAGCGTAGAGAAGACTAAATCTGGAAATATTTGTGATATTGTCATCCCGGTTGATGGTACAACATGTGGCGTCAACAAGTGGTGTCAAGATGGAAAATGCGTGATCAATCCAAATGGATCAGATTCCGCAACTACCTGCGAAGACAAAGTGGGTAAAGTACTGACTGTTGATGGGGTAAAGAGATCTTGCAAAGAGATCAAAAAAAATTATCCCTACATTTGCGAGCACGAATACTATTCAAGCTACTGTTGTAAGACTTGTCAGAAGGCAACCAGTTGACCAAAACAGGGAGCAGGAATGGAGACTTTAATGGTGCTTGTTTCATTCAGAACCTTTATGACATAAAAGTGACTTCTGACATTTATAACTTGGCTCGATCAGGTTGTTAAATGTCCAAATCGTTTAATTGATTATTAAAATTTGATTGTTTAAGAATCAGTGAATATTGACTTGTTCAAATTTTCAAATAAATTAATAATTATAATAATTAAATATACTTTGCATCACTCGCGGCAAAGGTGTTCGACTGTCGTATGATATCAGATTTATTTATTTGATTAAAGTACATTGTATAACTAGGGTGAATTGGGCTTCCACAAAAAGACTCGAAAGCAAAAACAAAACAGAAGAGGCTTCGATAAGCACGCAATCCTGTCATCTGATTCGCAAAAATCAAGATCCCGATAACGTTAGATATAGATAGTAGCATTTCCTTGTTTTTGAGTAACAAACCTTTTTATGAGTTGGGTTTTTTCCACCAAATGAGTTTCCAC |
| Unigene2342 | GAATGATTGTGGTATGAATTCGACAATAATTTATTTTTCAATCTTCGTTTATTTTTCTTCATGTATTTTATCCATATATACTAAAATAATCATGTGATGTGAAACAAGCTTTATTGATCAAATGAAGGTCCTTATTCGCATCCCATCATAAAGAGTTTAAATTTCCAATCGAAGAATATTTTCAGGGCTTGTAGCACAAGCCATTCCTGCAAGTGTATCTGGGCTCGCATTCGGAACAAGCAGGTCCAACTTTGAAAGGCTTGGCACCGCGATAATTTCCACCTGGACCGTAGTTGCAAACCATCAAGGTTCCACTGACTTTTCCTTCTTCCTTCTTTCCGGAACAGATGGTTGCGGCGCAACCAACGTATTCTGAAGCTGCCCAGACAACCTGAGTGTAATGTCCGCAAACTGCCCTGTCTCTACATTTGGTTTCGAAGTATTTATAGTCCTTCTTTTCGTTAAAGAAATTTTGTATTCCATCAACAGCATCGACCGACTTGGAGCTGCTCCAGTGTATGTTTTGGCCGACGTATCCGAAGTTGGTCTTTACACCAGTTGGATGTCCGTGCTTGAATTCGCAGCCGTCAGCCCACATGCTTGCAGTCTTAGCCAAAGTTTTATCCCATTTCATTTCCTTCATGTTACTTGAACCCTCTCCTCTCCTAAGCTTGTTGTGATGATCAAGAACACGCTGTTCATTTTTTTTAGACAACAACGTTCCAGATGCGAGGCACAATGTCGCAGCGATGAGAAAAAAGGTCTTCATCATCTCGATGTTCTGAAGGTTCAAATTATGCGTTTGAAATCGTGATTGAAATGTTTCAACTTTAAAAACAATCTCAATTATTAATTTTAGCAATTAATAATGCAATTCGATCACGTTTTTGACGTTGTAAACGGCG |
| Unigene3360 | CTCAAATAATGAAATAAAAGCAAATATGGTATTAGAGAACATAGGACACTTTTTTACTCAGTTCAAAAAACGTCTCTTAGTGTAATTAAGGAAATGCATTTTAAAAATTGGAAATATTTACAATTGATTTATGTTTTTAGTGTTCTAAAGGCAGTGGAAAAAAACAAATTATTGAAATTTGATCAATGCGTGGGTATATTTTGAGCCTCGAAGTCACAATATAAATACTTCATCGGTTCAGAAGACAATACCACTTAAACAGCGGCGGAACCAAAAATCAACAGAATGGCCGTATTTCTTTTGCAATTTTTCATACTGCTCTTAGCGTATCAAGCAAATTCAGACGAAATAAATGGTTTAGCCGCTGCCAACAATGATTTTGGACTGCAACTGTACAACAAGTTTGTGGAAACAGAACAAGGAAATATCATTTTCTCTCCTCTGAGTATTTCTCTTTCCTTGGCAATGACTTCGCTTGGAGCCAGAGGTCAGACATTGAAAGAAATTCGTCGTTCAACATTTACCAGTGGATTTCCAGAGGATGAAATTCATGAACAATTTCAACAAATTATGAAAGTCGTTCCAAGAAACGATGAAAATGAACAAATAAGCATCGCGAACAAATTGTATGCTCAGAAAGGCTTTGAAATTATTTCATCATTTCTTGAATCGTCTCAAAAATTCTATAAATCGGAACTTCAATCTGTTGACTTCAGCAATAACAAAAAAAGTGCTTCTCTAATTAACAACTGGGTATCTGAAATAACCAACAAGAAAATCACAGAGATAGTCAAACCCAATGATCTTACTAAACTTACTCGCCTTGTTCTCGTCAATGCCTTGTACTTCAAAGCTCAATGGGCTTCAGTTTTTAAGCAGCATTTAACGAAATCTCAAAATTTCTATATAACACCTAAAAATTCAGTAGAAACAGAATTTCTTCATGATTCAGCATCAAGATGTTCTTTTGGCGTGCATGAAACTCTCCACTTTGAAGTTTTAGAAAAAGCATTCAAATCACCTGAATTTGTATTTGGAATCATCCTTCCCGATCTGGCCAAAACATCACTAGATCAAGTCGAGAAAGGTTTGACGGCAGATGCACTAAAGTCTCTTGAATTAAAATCGATAATTGCAGACATTACCATTCCCAAATTTAAGTTCCAGTCAAGCTTTGACTTATCTGTTAATCTTAAAAAACTTGGAATCAATGATTTGTTTTCGGATAGAGCAGATCTGAGTGGGATAGAAGAATCAAAAGGTCTGTATGTTTCAAAGGCCTTACACAAAGCAGTTGTTGAAATTGATGAGAATGGTGCTGAGGCTGCTGCAGCAACAGCCATTGTAATTTCTACAAAATCAGCTTTCATACGTCGTCCTGTCATCAACTTCAGAGCTGATCATCCATTCCTATTTTATATCAAACACGTTGATACTGGTTCAATTCTTTTCCTTGGGAGATTTATTCAGCCTTGAAACCACCTATATGAGAAGTTTGTGTCATTGACATACATTATGATTTATGAACAATCAGGAAAGCATGATTATAACACATAATTTCTTAAAACTAAATGACGATTGTTATCAAAGTTATTGTTTTAAAAAATTAGTAAATTATCCACATTATTTTTATGGAAATAAAAAAATCCATTTATTAGTTTCTGC |
| CL4396.Contig2 | GGTTAACTATCTTATTACGTTTACTTCTAGTGCAGGACGGATTAAAGAATCTTCGGAATGGCCATTTGTCTTTTGCAGTTCTTCATTCTGTTTCTAGGATGTCAGGTAAATTCAGATACATCGGAGAAGTTAGTTTTTGAAAACAATGACTTTGCCTTGAAATTGTACAATAACTTGGTCGAGCAAAAGCAAACAAACGTAATATTCTCTCCTCTGAGTATTTCCCTCGGTTTGGCAATGACTTCGCTTGGAGCCAAAGGTCAGACTTTGAAAGAAATTCATTCGGCAATCGCTGGCAATTTTCCAAAGGATAAAATTGATGAAACGTTTCAACAAATCATGAGAGGAGTTCGTAGAAGCGAAACATTTGCCAAAGTCATCGTGGCAAACAAGTTGTACGCTCAGAAAGACTTTGACATTCTCTCGTCATTTAAAAAATCGTCTCAAAAAGTCTATGAATCGGGATTTCAATCTGTCGATTTCAGCAATAAGAAAGAAAGCGCTTCTCAAATTAACAACTGGGTGTCGGAAGAAACCGACGGAATAGTCAAAGACATAGTCAAACCAGAAGATCTTACCTCACCCACTAGTCTTGTTCTCGTCAGCACCTTGTACTTTAAAGCTCAATGGAATTCAGCTTTTGAGAAAGGTTCAACGACACGTCAGAAATTCTATGTTACACCTCAGAAATTTGTAGAAAAGGCTTTTCTTCATTCTCCGCTAACAGCAGTCTTTCACGGTCATATTAATAAAACTCTCCATTACGAGATTTTAGAAATACCTTTCACTTCAGGTGAATTCAGATTCGGGATCATCCTTCCCGACTTAAAGAAAAACCCACTCGATCAAGTCGAAAAATCACTGAAAGCAGATACATTTAAATCTCACGAATTAACTTCGCTAATGGCAAATGTCTTCATTCCCTCATTTAGAGTAGAATCGACCTTTGACTTATCAGACAATCTTAAAAAGCTTGGAATATCAAAATTGTTTGAGAAGGGTAAAGCAGATCTGAGCAGCTTAGGACAAACTAAAGGTATTCATGTTTCTAAGATCCTACACAAAGTCGTTCTTGGAATTAACGAGGAAGGTGCAGATACTGCTGCTGCAGCAACCGTCACATCTTCTGTCAAATCGGATCAGGAAAATCCTTTGGTTCGCATCAATTTCAGAGCTGACCATCCATTCCTGTTTTACATCATTGACATTGGTACGGGGTCATTAATTTACCTTGGGAGGTATTCCTCCAGTCCTTGAAGACGAGAGATTTTTGTCCTTGGTTATCACAATTGCATTAATATTTGGAAACACTCTGTTGAAATTCATTTAACTGATTACATC |
